# Between Aromatic and Quinoid Structure: A Symmetrical UV to Vis/NIR Benzothiadiazole Redox Switch

**DOI:** 10.1002/chem.202004009

**Published:** 2020-11-23

**Authors:** Philipp Rietsch, Sebastian Sobottka, Katrin Hoffmann, Alexey A. Popov, Pascal Hildebrandt, Biprajit Sarkar, Ute Resch‐Genger, Siegfried Eigler

**Affiliations:** ^1^ Institute of Chemistry and Biochemistry Freie Universität Berlin Takustraße 3 14195 Berlin Germany; ^2^ Institute of Chemistry and Biochemistry Freie Universität Berlin Fabeckstraße 34–36 14195 Berlin Germany; ^3^ Department 1, Division Biophotonics Bundesanstalt für Materialforschung und -prüfung (BAM) Richard Willstätter Straße 11 12489 Berlin Germany; ^4^ Leibniz Institute for Solid State and Materials Research Helmholtzstraße 20 01069 Dresden Germany; ^5^ Chair of Inorganic Coordination Chemistry Institute of Inorganic Chemistry University of Stuttgart Pfaffenwaldring 55 70569 Stuttgart Germany

**Keywords:** benzothiadiazoles, fluorescence, molecular switches, quinones, spectroelectrochemistry

## Abstract

Reversibly switching the light absorption of organic molecules by redox processes is of interest for applications in sensors, light harvesting, smart materials, and medical diagnostics. This work presents a symmetrical benzothiadiazole (BTD) derivative with a high fluorescence quantum yield in solution and in the crystalline state and shows by spectroelectrochemical analysis that reversible switching of UV absorption in the neutral state, to broadband Vis/NIR absorption in the 1st oxidized state, to sharp band Vis absorption in the 2nd oxidized state, is possible. For the one‐electron oxidized species, formation of a delocalized radical is confirmed by electron paramagnetic resonance spectroelectrochemistry. Furthermore, our results reveal an increasing quinoidal distortion upon the 1st and 2nd oxidation, which can be used as the leitmotif for the development of BTD based redox switches.

## Graphical abstract

**The quinoidial distortion** induced in benzothidiazoles upon oxidation was used in this work to design an absorption redox switch. Using spectroelectrochemical analysis, the absorption, fluorescence and localization of the radical were investigated during the redox processes. The symmetrical benzothiadiazole derivative can thus be used as a UV to Vis/NIR absorption switch or as a fluorescence ON/OFF switch. With deeper understanding of the quinodial distortion, it can furthermore be used as the basis for development of other small organic redox switches.

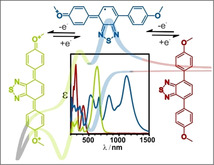

Changing the color (or opacity) of a material by the application of voltage is known as electrochromism. This effect is commonly used to tint rear‐view mirrors in cars or to control heat transfer through surfaces (smart windows). To date, most of the employed electrochromic materials are metal oxides, for example, WO_3_, with only some organic exceptions, mainly viologens.^[1]^

Reversibly switching the absorbance and/or the fluorescence by redox processes bears a high potential for future applications in light harvesting, sensors, and medical diagnostics. Small molecular compounds have been in the focus of research as functional dyes, probes and sensors for neutral and ionic analytes and as active components in organic light‐emitting diodes (OLEDs), optical devices, and lasers.^[2]^ However, few of these investigations employ spectroelectrochemical methods, and most of the known optical redox switches consist of complex molecular structures, which employ tetrathiafulvalenes or quinone motifs as redox systems.^[3]^ Bard et al. investigated the redox behavior of symmetrical dithienylbenzothiadiazoles (BTD) and focused on the electrochemiluminescence (ECL) in solution and solid films.^[4]^ Recently, Liang et al. presented a symmetrical triphenylamine substituted BTD, with one reversible oxidation and reduction process, strong ECL, and used it as a sensor for dopamine.^[5]^ However, BTDs have not been considered for absorption or color switching through redox processes. We have recently demonstrated that switching the absorption between UV and Vis/NIR by redox processes seems to be feasible with small molecule BTD derivatives.^[6]^ These derivatives (Figure 1 A) however, lacked the desired reversibility of the redox processes.

**Figure 1:**
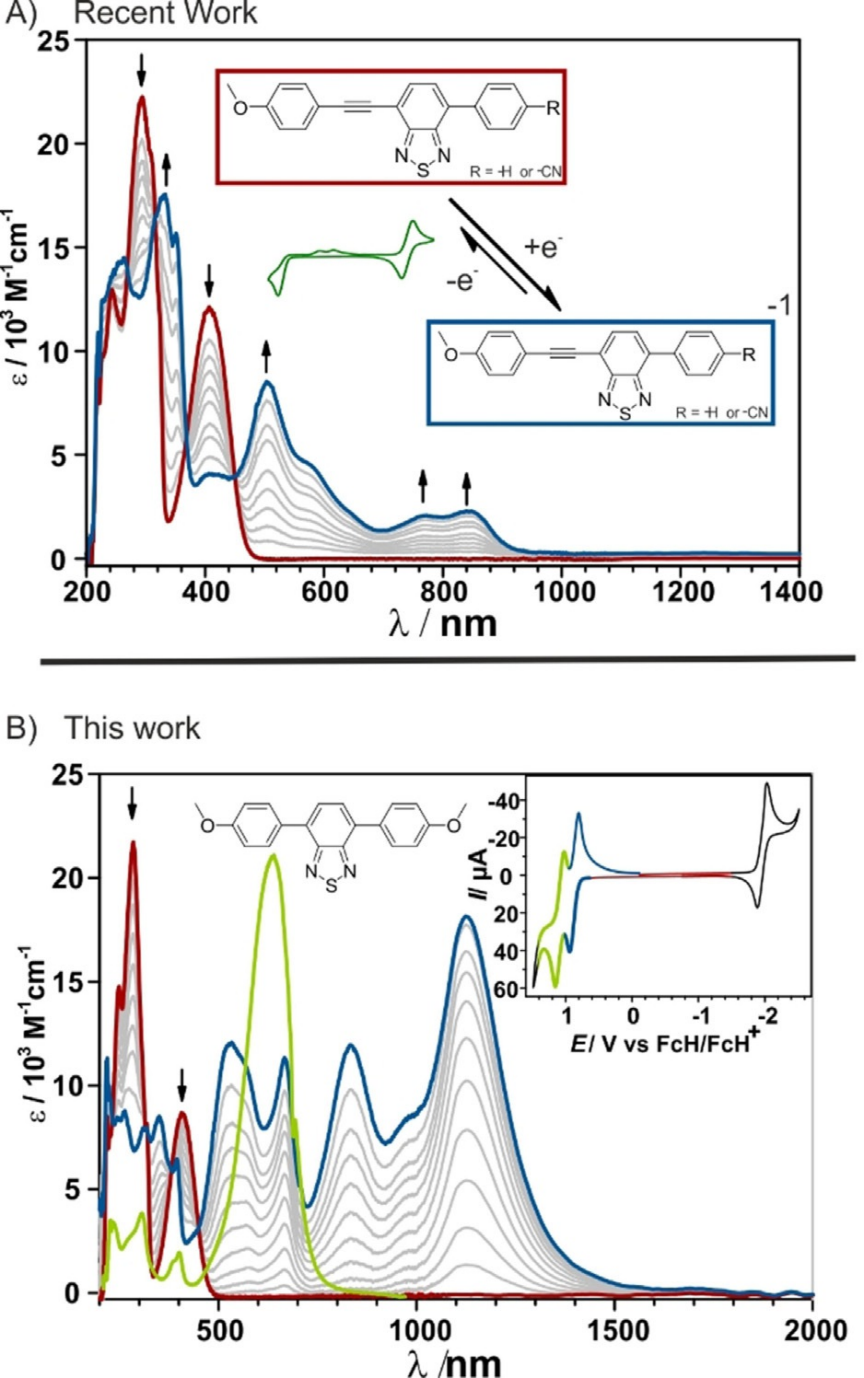
Spectroelectrochemical investigations of A) recent work: Only partly reversible, reductive redox switching between UV and Vis/NIR absorption realized with benzothiadiazoles featuring an alkyne moiety. B) This work: shows reversible redox switching between UV, Vis/NIR and Vis absorption with two fully reversible oxidative processes.

Herein, we investigate the fundamental properties of a symmetrical benzothiadiazole derivative by a combination of spectroscopy, electrochemistry, spectroelectrochemistry and theoretical calculations. Spectroelectrochemical measurements (CV‐UV/Vis/NIR) of this highly emissive molecule, that has a fluorescence quantum yield of up to 93 %, show that its absorption can be switched between the UV (neutral form), to multi band Vis/NIR absorption (1st Ox.), to a sharp single band Vis absorption (2nd Ox.) and back to UV. Electron paramagnetic resonance spectroelectrochemical measurements (EPR‐SEC) reveal the formation of a delocalized radical after the first oxidation and DFT calculations indicate an increasing quinoidal distortion upon the first and second oxidation, which was not considered before.

We started the synthesis of the symmetrical di‐methoxyphenyl BTD **1** from *o*‐phenylenediamine following a published procedure (Figure 2 A).^[7]^ The Suzuki reaction of 4,7‐dibromo‐2,1,3‐benzothiadiazole with 2.2 equiv. of (4‐methoxyphenyl)boronic acid and 0.1 equiv. of Pd(PPh_3_)_4_ afforded **1** in a yield of 91 %.

**Figure 2:**
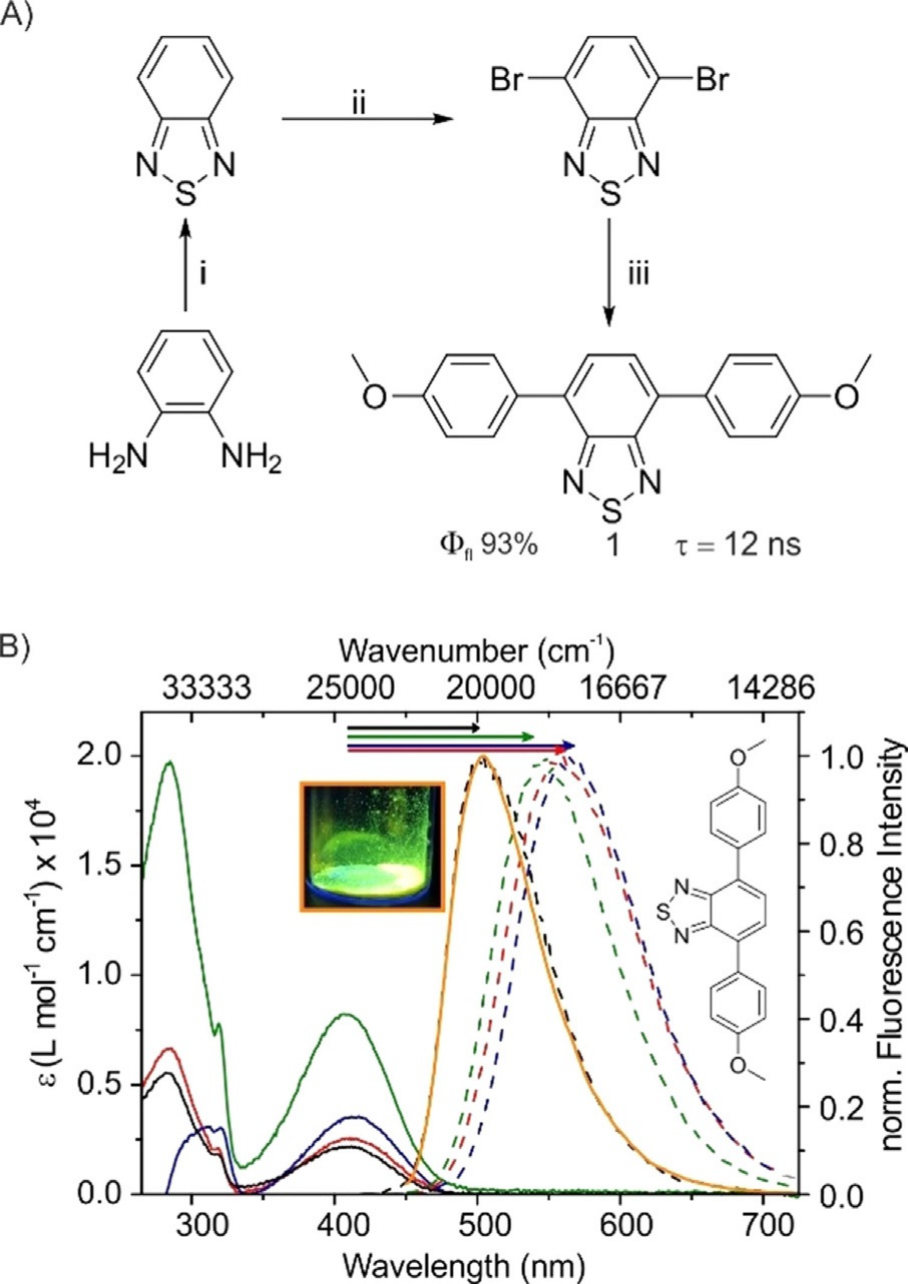
A) Synthetic route to symmetrical benzothiadiazole **1** starting from *ortho*‐phenylenediamine. i) SOCl_2_, DCM, 5 h, reflux, yield=93 %. ii) HBr, Br_2_, yield=76 %. iii) THF/H_2_O, 2.2 equiv. of (4‐methoxyphenyl)boronic acid, K_2_CO_3_, Pd(PPh_3_)_4_, yield=91 %. The fluorescence quantum yield (Φ_fl_) and fluorescence lifetime (τ) in DCM is given. B) Absorption spectra (solid) and normalized fluorescence spectra in solution (dotted, exc. at 400 nm, *n*‐hexane= black, DCM=green, DMSO=blue, EtOH=red) and the solid state (orange, exc. at 355 nm) of **1**. The dye concentration was 10^−5^ m. The inset shows the crystalline sample under irradiation with light of 366 nm.

Subsequently, we performed a photophysical characterization of BTD **1** in four solvents of varying polarity and proticity, namely *n*‐hexane (apolar and aprotic), dichloromethane (DCM; medium polarity, aprotic), dimethylsulfoxide (DMSO; polar and aprotic), and ethanol (EtOH; polar and protic) as well as in the solid state.

The absorption and normalized fluorescence spectra of **1** are shown in Figure 2 B. In DCM, the molar absorption coefficient (*ϵ*) of the π–π* transition at 280 nm is 20 161 m ^−1^ cm^−1^ and about 8611 m ^−1^ cm^−1^ for the HOMO–LUMO transition, very likely the S_0_‐S_1_, at 400 nm (Table 1).^[8]^ The fluorescence spectra show a positive solvatochromism, indicated by the arrows in Figure 2 B, of about 82 nm (*n*‐hexane: 473 nm vs. DMSO: 555 nm). That is characteristic for BTDs as well as compounds with intramolecular charge transfer (ICT) character, such as push‐pull coumarins, and indicates an increasing dipole upon excitation.^[9]^ The fluorescence spectra in the solid state (Figure 2 B, orange curve) resemble the emission spectrum in *n*‐hexane.

**Table 1:** Molar absorption coefficients (*ϵ*) of the two absorption maxima (see Figure 2 B), fluorescence quantum yields, determined absolutely with an integrating sphere setup (see Supporting Information), and fluorescence lifetime of compound **1** in solvents of different polarity and proticity. The values for the normalized Dimroth–Reichardt Parameter E_T_^N^ were taken from ref. [9a].

E_T_^N^	Solvent	*ϵ* [M^−1^ cm^−1^]	*φ* [%]	τ [ns]
0.006	*n*‐hexane	5521/2166	83	8.92
0.309	DCM	20 161/8611	93	11.97
0.444	DMSO	3090/3511	93	11.74
0.654	EtOH	6669/2557	64	10.06
	solid	–	66	–

The Φ_fl_ of **1** is maximal in DCM and DMSO with values of 93 %, and minimal in EtOH with a value of 64 %. We proved that the compound follows the Lambert–Beer law, even in EtOH, where it is least soluble by two dilution series, one in DCM and one in EtOH (Figure S20). This reduction in Φ_fl_ is therefore ascribed to hydrogen bonding interactions with the protic solvent and the BTD core and the ICT character of the dye's emission in this solvent, as shown before.[^6^, ^9b^, ^10^] The fluorescence lifetimes (τ) of about 9 ns in *n*‐hexane and up to 12 ns in DCM are relatively long compared to other BTD, which possess lifetimes in the range of 2–10 ns.^[6]^

The push‐pull BTDs, which we investigated in another study^[6]^ showed an 50 % increase of Φ_fl_ from polar, protic EtOH to apolar, protic 1‐decanol (1‐dec). In contrast, the Φ_fl_ of the symmetrical BTD **1** increases by only about 9 % from EtOH to 1‐dec (Table S4). As demonstrated by our measurements in different mixtures of EtOH and polyethylene glycol (PEG), which have a constant polarity and an increasing viscosity, the increase of Φ_fl_ in primary alcohols can be assigned to a change in viscosity (1‐dec is about 12 times more viscous than EtOH (Table S2)). This difference between symmetrical and asymmetrical push‐pull BTDs goes hand in hand with differences in their ground state and excited state dipole moments, and the resulting charge separation and charge distribution in the respective intramolecular charge transfer (ICT) and the locally excited (LE) states. The population of the ICT state is preferred in polar solvents for structures, which enable stronger charge separation, for example, push‐pull systems.[^9b^, ^10^, ^11^] That is in agreement with our finding, that the change of dipole moment upon excitation, derived from the Lippert–Mataga plot, of **1** is 12.4 Debye and thus smaller compared to our push‐pull derivatives with values up to 18.0 Debye (Figure S6).

We performed cyclic voltammetry (CV) as well as spectroelectrochemical (UV/Vis/NIR and electron paramagnetic resonance (EPR)) measurements of **1** in a 0.1 m Bu_4_NPF_6_ solution of DCM. In the cyclic voltammogram, the compound shows one reversible reduction process at −1.96 V and two electrochemically reversible one‐electron oxidation processes at +0.87 V and +1.09 V (Figure 3 A and Table S5), in agreement with earlier investigations of a symmetrical dithienyl BTD derivative.^[4]^

**Figure 3:**
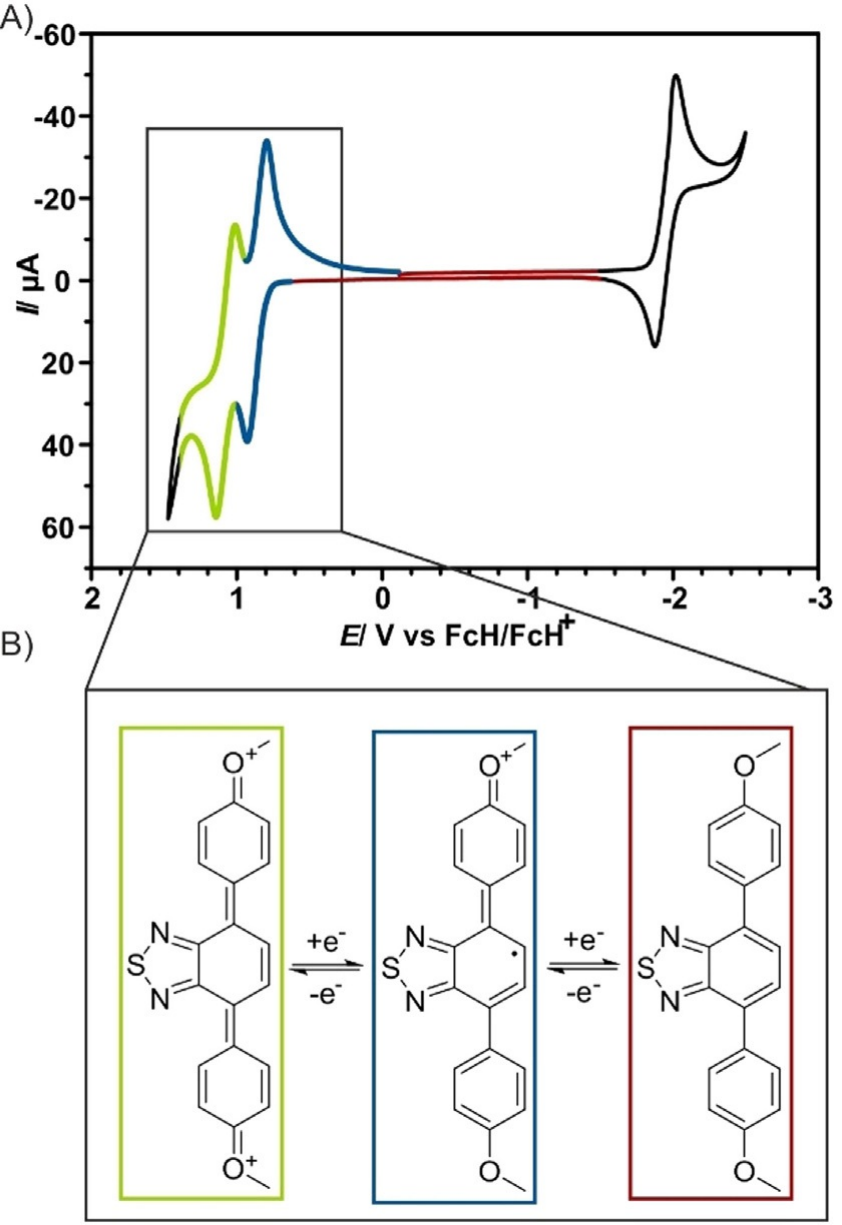
A) Cyclic voltammogram of **1** measured at 100 mV s^−1^ in 0.1 m Bu_4_NPF_6_ solution of DCM in oxidative direction first (purple) and reductive direction first (black). B) Proposed semiquinone and quinone structures of **1^+^** and **1^2+^**, which are supported by DFT calculations at D3‐BP86/def2‐TZVP level of theory.

The push‐pull BTDs recently investigated by us bear all an alkyne moiety and undergo irreversible redox processes.^[6]^ However, BTD with push‐pull systems have been published, which show reversible redox processes.^[12]^ We therefore conclude, that the alkyne moiety induces a follow‐up reaction leadings to this irreversibility, which is not favorable for the design of optical redox switches. The reversibility of compound **1** supports this assumption. Furthermore, and in contrast to the push‐pull BTDs, the redox responses of **1** are independent of the scan direction, and the reduction and 2nd oxidation are incoherent (Figure S8A). This is in accordance with other electrochemical investigations of BTDs.^[13]^ We employed UV/Vis/NIR‐spectroelectrochemistry (SEC), coupling cyclic voltammetry with UV/Vis/NIR spectroscopy, to probe the chemical reversibility of the redox processes and the follow‐up reactions of **1**, **1^+^**, and **1^2+^**. This was done using OTTLE (optically transparent thin layer electrochemical) cells filled with a defined starting concentration of **1** of about 1×10^−4^ m in DCM.^[14]^ We furthermore undertook TD‐DFT calculations at the B3LYP/def2‐TZVP level to rationalize the electronic structure of the redox states of **1** (Figure S13–S17 and Table S8–S15).

The initial visible band of **1** at 408 nm is attributed to a π–π* transition from the π‐systems of the electron‐rich methoxy‐phenlyene moieties to the central electron deficient BTD π‐system. The spectrum is well reproduced, however slightly redshifted as expected for this level of theory (Figure S13). The absorption spectrum of **1^+^** shows four distinct bands in the visible and NIR‐region, at 531, 666, 832, and 1124 nm (Figure 4 A) with molar absorption coefficients of 10 000–20 000 m ^−1^ cm^−1^. Similar spectra with comparable bandwiths are observed for related semiquinone anion radicals, which are isolobal to diamino radical cations and the here investigated BTD derivative, and where interpreted as the vibrational fine structure of the radical compound.^[15]^ In fact, the results of the geometrical optimization (D3‐BP86/def2‐TZVP) for **1**, **1^+^** and **1^2+^** predict an increasing quinoidal distortion of the aromatic system upon oxidation, in analogy to its isolobal congeners (Figure 3 B). Whereas the absorption spectra of **1^2+^** resembles the calculated one (Figure S15), the calculated spectrum of **1^+^** differs from the measured one (Figure S14 and Table S11). Calculations which consider also the solvent molecules and hence the solvation shell of **1^+^** showed no improvement (Figure S16A). However, if the multiple transitions of **1^+^** are indeed interpreted as the vibrational fine structure, then the calculated transitions lie in between the two measured couples (531+666 and 832+1124 nm) respectively. The UV/Vis spectrum of **1^2+^** is dominated by an intense transition at 654 nm, which is mostly assigned to π–π* transitions of the oxidized methoxyphenyl moieties and to a charge transfer from the BTD π‐system to the adjacent phenyl rings (Figure 5 A), which are electron‐deficient after double oxidation.

**Figure 4:**
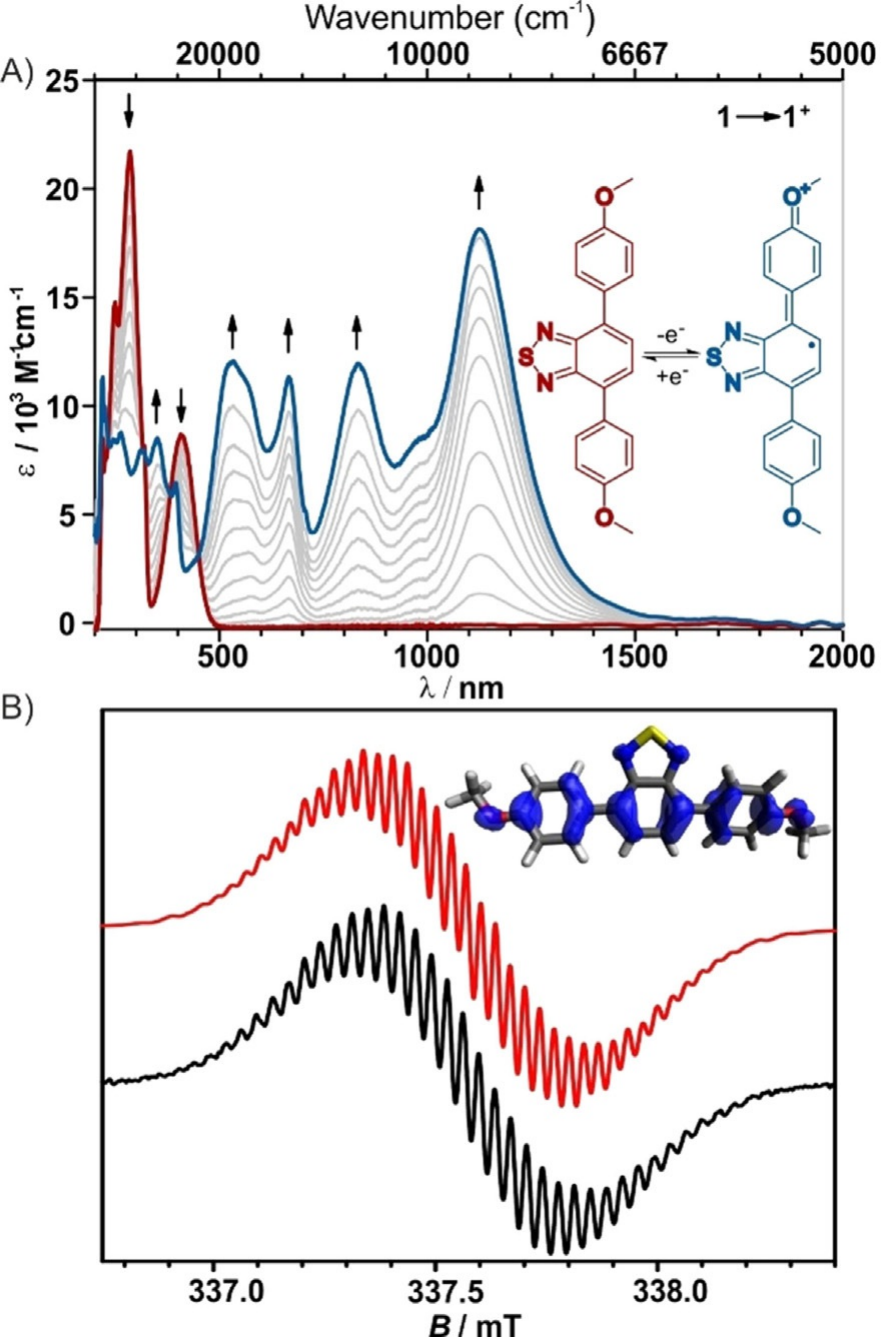
A) Absorption spectra of **1** before (red) and after the first oxidation (blue). The proposed structure of NIR absorbing **1^+^** species is shown in the inset in blue. B) EPR spectra of **1** measured after oxidation (black) and simulated (red). Inset: Spin density of **1^+^** calculated at the PBE0/IGLO‐III level.

**Figure 5:**
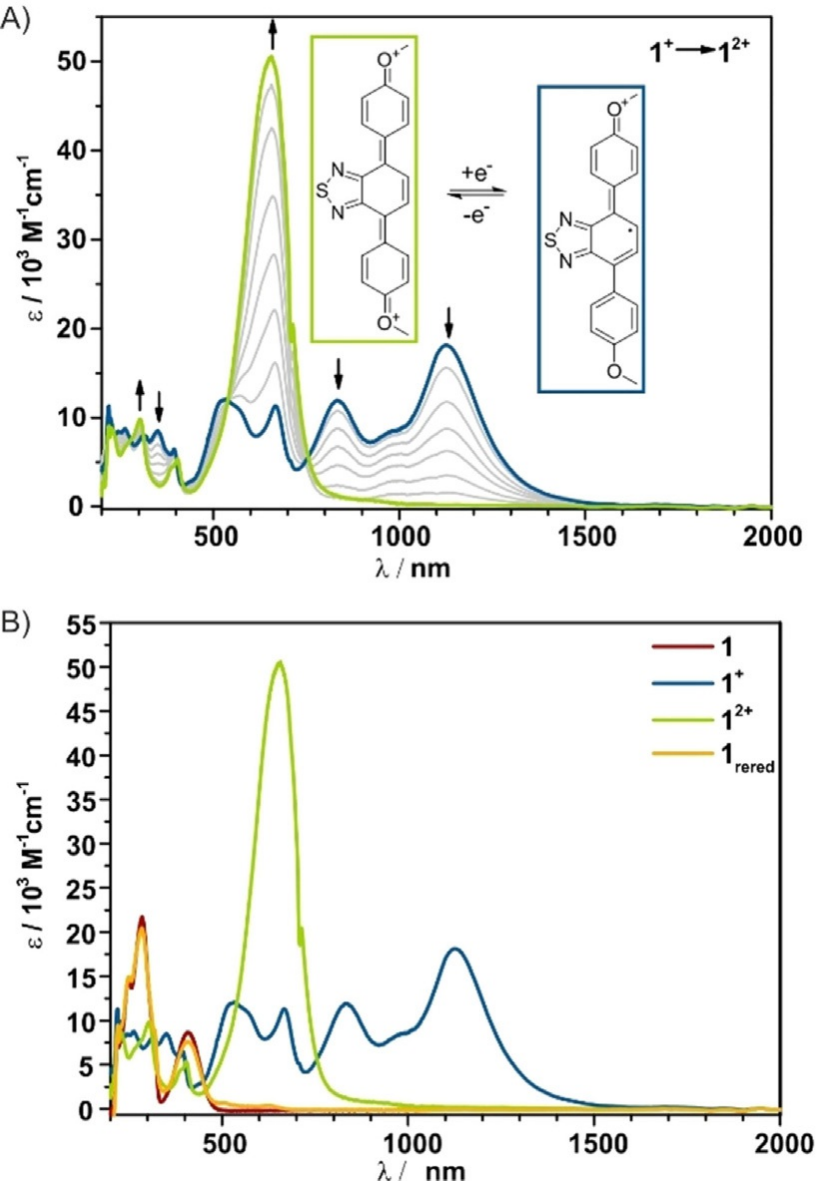
A) Absorption spectra of **1^+^** (blue) and **1^2+^** (green). The proposed structures in a rectangle of the respective color are in the inset. B) Absorption spectra of **1** in the neutral state (red), after 1st oxidation (blue), after the 2nd oxidation (green) and after re‐reduction (orange).

In recent studies, electrochemiluminescence (ECL) was observed for symmetrical BTDs in thin films.[^4b^, ^5^] Therefore, we measured the fluorescence intensity during the oxidation. The strong emission of the neutral compound (excitation at 405 nm) is reduced upon oxidation and restored upon re‐reduction (Figure S18). However, excitation at the absorption maximum of **1^2+^** (638 nm), yields no fluorescence signal (Figure S19). We therefore conclude, that fluorescence ON/OFF redox switching at an excitation wavelength of 405 nm is possible.

Interestingly, the reduction of **1** appears to be electrochemically reversible, however the initial absorption spectrum is not obtained after a complete reductive cycle in UV/Vis/NIR‐SEC (Figure S10). Upon reduction, very broad Vis and NIR bands emerge without distinguishable peaks (Figure S10A), which do not change, even after re‐oxidation. This may indicate the occurrence of electro‐polymerization at the electrode. Since the SEC experiments take place on a considerably longer time scale (1–3 minutes) than the CV experiments to ensure full conversion, the electro‐generated species have more time to participate in subsequent chemical reactions.

The chemical stability and reversibility of the redox couples **1/1^+^** and **1^+^/1^2+^** were determined to be 95 % and verified by spectral recovery in an anodic/cathodic scan reversal (Figure 5 B).

The radical cation **1^+^** was further investigated by EPR spectroscopy. For this purpose, the radical was generated in situ via electrolysis (Figure 4 B black curve). The radical shows an isotropic signal at room temperature with a g‐value of 2.0028 and a rich hyperfine structure. This suggests a strong delocalization of the unpaired electron over the whole π‐system, which is reproduced by our DFT calculations (PBE0/IGLO‐III) suggesting a delocalized spin population (see Figure 4 B red curve). It was possible to simulate the EPR spectrum by considering hyperfine coupling to two ^14^N nuclei (*I*=1) and to 10 ^1^H nuclei (*I*=1/2 ) with different coupling constants (see SI for details).

We developed a reversible organic redox switch, which enables absorption or color switching between the UV, Vis/NIR, and Vis, as well as fluorescence ON/OFF switching. The EPR spectrum of the 1st oxidized state unravelled a strong delocalization of the unpaired electron over the whole π‐system, which is reproduced by our DFT calculations and is accompanied by increasing quinoidal distortion, which can be used as the leitmotif for the development of BTD‐based redox switches. This symmetrical benzothiadiazole is easily accessible in three synthetic steps and the compound shows a high fluorescence quantum yield of more than 90 % in solution and 66 % in the solid state.

## Conflict of interest

The authors declare no conflict of interest.

## Supporting information

As a service to our authors and readers, this journal provides supporting information supplied by the authors. Such materials are peer reviewed and may be re‐organized for online delivery, but are not copy‐edited or typeset. Technical support issues arising from supporting information (other than missing files) should be addressed to the authors.

Supplementary
