# Supplementary material for: Between Aromatic and Quinoid Structure: A Symmetrical UV to Vis/NIR Benzothiadiazole Redox Switch
Source: Chemistry. 2020 Nov 23;26(72):17361–5. doi: 10.1002/chem.202004009 (PMC7839704; doi:10.1002/chem.202004009)
Supplement: Supplementary file 1 — Supplementary [file CHEM-26-17361-s001.pdf]

Chemistry–A European Journal

Supporting Information

Between Aromatic and Quinoid Structure: A Symmetrical UV to Vis/NIR Benzothiadiazole Redox Switch

Philipp Rietsch+,[a] Sebastian Sobottka+,[b] Katrin Hoffmann,[c] Alexey A. Popov,[d] Pascal Hildebrandt,[a] Biprajit Sarkar,[b, e] Ute Resch-Genger,*[c] and Siegfried Eigler*[a]

Table of contents

1. General Information 2
2. Synthetic procedure 3

4-7-bis(4-methoxyphenyl)benzo[c][1,2,5]thiadiazole (1)........ 3

3. Fluorescence Lifetimes Measurements 6
4. Measurements in primary alcohols and mixtures of ethanol/polyethylene glycol 7
5. Lippert-Mataga Plot 9
6. Solid-state Fluorescence Measurements 10
7. Cyclic voltammetry measurements 11
8. UV/Vis/NIR-spectroelectrochemistry 12
9. EPR spectroelectrochemistry of **1** 14
10. TD-DFT Calculations for 1 (singlet) 15
11. TD-DFT for 1^{+} (doublet) 16
12. TD-DFT for 1^{2+} (singlet) 17
13. TD-DFT for 1^{+} with explicit solvent molecules 18
14. TD-DFT for 1^{-} (doublet) 19
15. In situ fluorescence measurements during oxidation and re-reduction 20

1. General Information

All reagents were purchased from commercial sources and used without further purification. Dry solvents were purchased from Acros Organics. ALUGRAM Xtra SIL G/UV_{254} plates by Macherey-Nagel were used for thin-layer chromatography. Isolation of products by chromatography was performed with silica from Macherey-Nagel Silica 60 M (0.04-0.063 mm). NMR spectra were recorded on a JOEL ECX 400 (^{1}H 400 MHz, ^{13}C 101 MHz), JEOL Eclipse+ 500 (^{1}H 500 MHz, ^{13}C 126 MHz) and BRUKER AVANCE 700 (^{1}H 700 MHz, ^{13}C 176 MHz) spectrometer at 25 °C. The chemical shifts δ are calibrated on the respective solvent peak as internal standard. All shifts are reported in ppm and NMR multiplicities are abbreviated as s (singlet), d (duplet), t (triplet), m (multiplet). Coupling constants J are reported in Hz. UV/Vis spectra were recorded on a Cary 50 Bio photospectrometer (Varian). Fluorescence spectra were recorded on a LS 50 B luminescence spectrometer from PerkinElmer. UV/Vis and Fluorescence spectra were measured in quartz glass cuvettes with 1 cm path length. IR Spectra were recorded on a FT/IR 4100 spectrometer from JASCO. Elemental analysis was performed on an VARIO EL from Elementar.

Photoluminescence quantum yields (Φ_{fl}) were determined absolutely with an integrating sphere setup from Hamamatsu (Quantaurus-QY C11347-11). All Φ_{fl} measurements were performed at 25°C using special 10 mm x 10 mm long neck quartz cuvettes from Hamamatsu. Values below 1% quantum yield are not reliable in the measurement setup and are therefore given as < 1%.
The fluorescence lifetime (τ), the average time in which the fluorophore is in an excited state before it relaxes to the ground state, was recorded on a fluorometer FLS 920 (Edinburgh Instruments) equipped with a Hamamatsu R3809U-50 (range 200–850 nm, response width <25 ps), Multi-Channel Plate (MCP) detector, Czerny-Turner double monochromators and either a supercontinuum laser (Fianium SC400-2-PP) or a Edinburgh Instrument EPLED-330 (picosecond pulsed light emitting diode) for excitation at 375 nm, or a Edinburgh Instrument EPL-375 (picosecond pulsed diode laser) for excitation at 330 nm. All the measurements were performed at T = 298 K using 10 mm _ 10 mm quartz cuvettes from Hellma GmbH always filled with 2 mL of solvent or dye solution. Before each measurement, the instrument response function (IRF) was measured. The lifetime measurements were analysed with Edinburgh Instruments FAST Software and fitted with a reconvolution fit. All the lifetimes could be evaluated mono, bi- or tri-exponentially with a reduced X^2 between 0.8 and 3.0.
The fluorescence spectra of the crystals in the solid-state and microscopic images were recorded with an Olympus FluoView FV1000 (Olympus GmbH, Hamburg, Germany). For UV excitation, a DPSS Cobolt Zouk® (355 nm; 10 mW), and for transmission imaging an additional multiline argon ion laser (30 mW, 488 nm) were used as excitation sources, which were reflected by a beamsplitter (BS 20/80) and focused onto the sample through an Olympus objective UPLSAPO 10X (numerical aperture N.A. 0.40). The emitted photons were recollected with the same objective and focused onto a PMT. Emission signals were detected in a wavelength range between 460 nm and 700 nm with spectral resolution of 5 nm and a step width of 2 nm. The spatial resolved fluorescence spectra are raw spectra, not specifically corrected for the wavelength-dependent spectral responsivity of the detection system of the microscope.

Electrochemistry

Cyclic voltammograms were recorded with a PAR VersaStat 4 potentiostat (Ametek) or a VersaStat 4 by working in anhydrous and degassed dichloromethane (99.8 % extra dry) distilled from CaH_2 with 0.1 M NBu_4PF_6 (dried, > 99.0 %, electrochemical grade) as electrolyte. A three-electrode setup was used with a glassy carbon, gold or platinum working electrode, a coiled platinum wire as counter electrode, and a coiled silver wire as a pseudoreference electrode. The ferrocene/ferrocenium couple was used as internal reference.

UV/Vis/NIR spectroscopy and spectroelectrochemistry

UV/Vis/NIR spectra were recorded with an Avantes spectrometer consisting of a light source (AvaLight-DH-S-Bal), a UV/Vis detector (AvaSpec-ULS2048), and an NIR detector (AvaSpec-NIR256-TEC). Spectroelectrochemical measurements were carried out in an optically transparent thin-layer electrochemical (OTTLE) cell (CaF_2 windows) with a platinum-mesh or a gold-mesh working electrode, a platinum-mesh counter electrode, and a silver-foil pseudoreference electrode.[1] The OTTLE cell with the gold working electrode was build analogous to the one with the platinum working electrode (100 mesh woven from 0.064 mm diameter wire; 99.99% (metals basis)). The molar extinction coefficients of the electrochemically produced oxidized (radical cations) and reduced (radical anions) intermediates were derived for samples containing a known concentration of the BDTs **1-4** which was about $1x10^{-4}$ M.

Electron paramagnetic resonance spectroelectrochemistry

EPR spectra at X-band frequency (ca. 9.5 GHz) were obtained with a Magnettech MS-5000 bench top EPR spectrometer equipped with a rectangular TE 102 cavity. The measurements were carried out in synthetic quarz glass tubes. For EPR spectroelectrochemistry a three-electrode setup was employed using two teflon-coated platinum wires (0.005” bare, 0.008” coated) as working (or a teflon-coated gold wire (0.003” bare, 0.0055” coated) as working electrode) and counter electrode and a Teflon-coated silver wire (0.005” bare, 0.007” coated) as pseudoreference electrode.

Computational Details

The program package ORCA 4.0.1 was used for all DFT calculations.[2] Starting from the molecular structure obtained from X-ray diffraction geometry optimizations were carried out using the BP86 method as developed by Grimme and co-workers[3] and no symmetry restrictions were imposed during the optimization. Subsequent single-point calculations were performed on the optimized geometries using the B3LYP functional.[4] All calculations were run with empirical Van der Waals correction (D3).[5] The restricted and unrestricted DFT methods were employed for closed and open shell molecules respectively unless otherwise stated. Convergence criteria were set to default for geometry-optimization (OPT), and tight for SCF calculations (TIGHTSCF). Relativistic effects were included with the zeroth-order regular approximation (ZORA).[6] Triple- ζ –valence basis sets (TZVP-ZORA)[7] were employed for all atoms. Calculations were performed using resolution of the identity approximation[8] with matching auxiliary basis sets[9] for geometry optimizations and numerical frequency calculations and the RIJCOSX (combination of the resolution of the identity and chain of spheres algorithms) approximation for single point calculations using the B3LYP functional, where applicable.[8] Low-lying excitation energies were calculated with time-dependent DFT (TD-DFT). Solvent effects were taken into account with the conductor-like polarizable continuum model (CPCM).[10] For all calculations. Spin densities were calculated according to the Löwdin population analysis.[11] The absence of imaginary frequency confirmed that the optimized geometries represent local minima. Spin densities and difference densities were visualized with the modified Avogadro 1.2.0 program with extended ORCA support.[12] Calculations of EPR parameters and spin density evaluations were carried out with the B3LYP functional and the IGLO-III basis set.[13]

2. Synthetic procedure

The synthesis of **1** was done as depicted in Figure S 1. The procedure to compounds **2** and **3** were done according to literature and the spectroscopic results were in accordance with literature.[14] Taking compound **7**, the reaction conditions of Park *et al.* were used to synthesize compounds **1**.[15] The numbers of the compounds were chosen for an easier understanding in the paper.

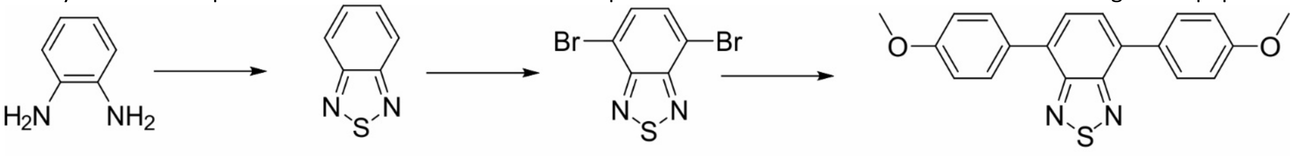

2 3 1

Figure S 1: Synthetic route towards benzothiadiazole derivatives **1**.

4-7-bis(4-methoxyphenyl)benzo[c][1,2,5]thiadiazole (1)

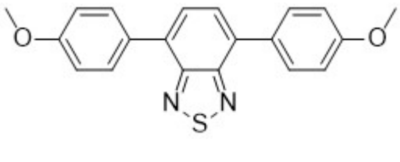

Under argon atmosphere, 4,7-Dibromobenzo[c][1,2,5]thiadiazole (300.00 mg, 1.02 mmol, 1 eq.), (4-methoxyphenyl)boronic acid (341.20 mg, 2.25 mmol, 2.2 eq.), K_2CO_3 (1.41 g, 10.21 mmol, 10 eq.) and $Pd(PPh_3)_4$ (94.40 mg, 0.08 mmol, 0.1 eq.) were stirred at 70 °C in a degassed mixture of tetrahydrofuran (15 mL) and water (5 mL) for 24 hours. The mixture was cooled to room temperature, diluted with water (10 mL). The suspension was extracted with dichloromethane (3 x 15 mL). The united organic phases were dried over $MgSO_4$, filtered and the solvent was removed under

reduced pressure. The crude product was purified by column chromatography with dichloromethane/hexane (1/1) to afford 4-7-bis(4-methoxyphenyl)benzo[c][1,2,5]thiadiazole (322.50 mg, 0.93 mmol, 91%) as a bright yellow solid.

^{1}H NMR (500 MHz, $CDCl_3$, RT): δ (ppm) = 7.92 (dd, ^3J = 8.5 Hz, ^5J = 1.5 Hz, 4H), 7.72 (d, J = 0.5 Hz, 2H), 7.08 (dd, ^3J = 8.5 Hz, ^5J = 1.5 Hz, 4H), 3.90 (s, 6H).

^{13}C NMR (126 MHz, $CDCl_3$, RT): δ (ppm) = 159.86, 154.31, 132.44, 130.47, 130.10, 127.49, 114.19, 55.49.

FT-IR (ATR) ṽ (cm^{-1}): 3051 (vw), 3041 (vw), 3010 (vw), 2989 (w), 2960 (w), 2932 (w), 2836 (w), 1605 (m), 1552 (w), 1513 (m), 1480 (m), 1457 (m), 1435 (m), 1343 (w), 1278 (m), 1248 (s), 1176 (s), 1114 (s), 1029 (s), 934 (w), 888 (m), 826 (s), 796 (s)

UV/Vis (DCM) λ_{max} nm (ε [$Lmol^{-1}$ cm^{-1}]): 285 (20161), 408 (8611)

Fluorescence (DCM) λ_{max} nm (ϕ_{Fl}): 546 (93)

MS (EI): m/z = 333.06 (57), 348.084 $[M]^{\cdot+}$ (100), 349.088 (41), 350.09 (13)

EA: $C_{20}H_{16}N_2O_2S$, calc.: C, 68.95; N, 8.04; H, 4.63; S, 9.20 meas.: C, 69.08; N, 7.45; H, 4.80; S, 9.72

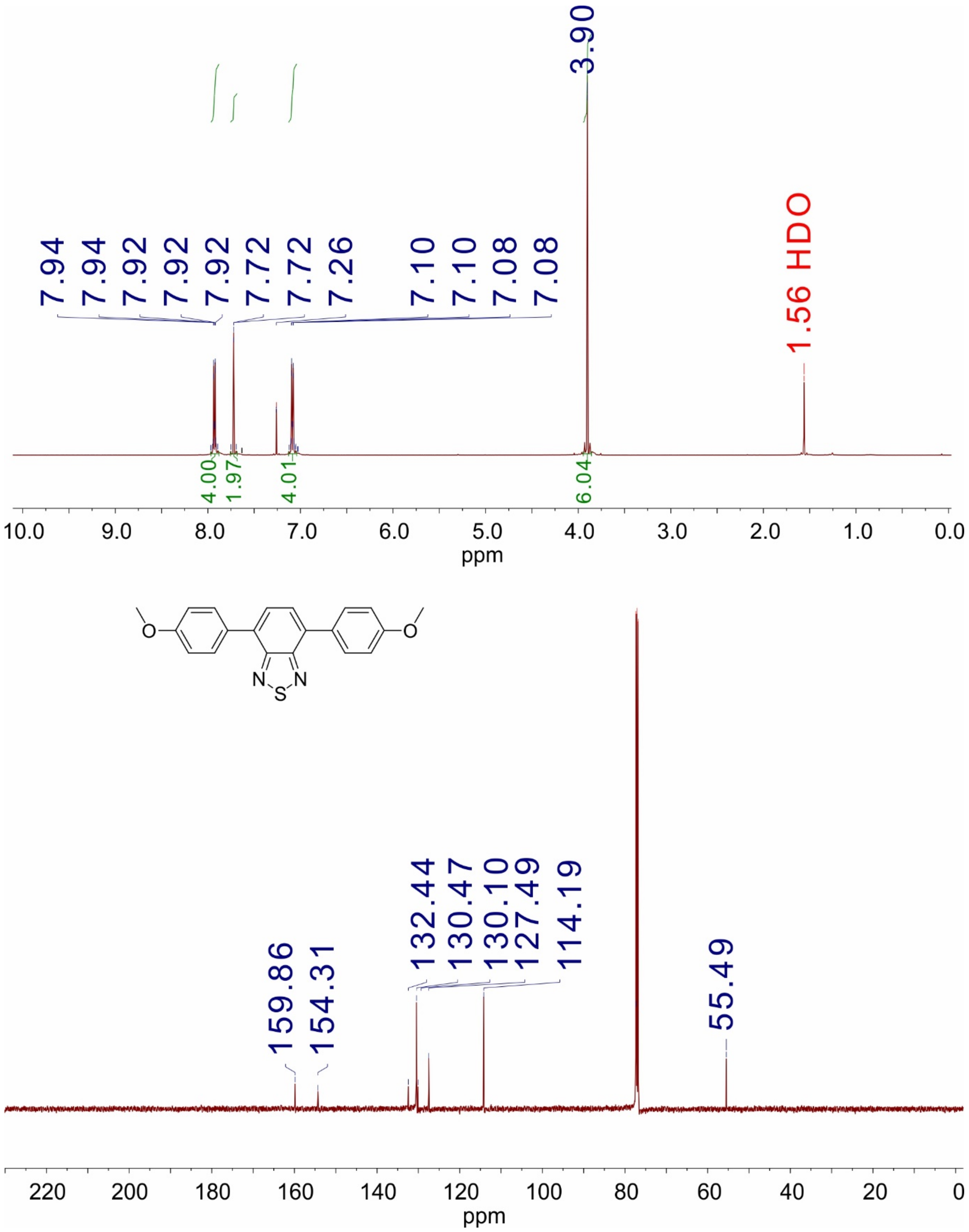

Figure S 2: ^{1}H -NMR spectra (top) and ^{13}C -NMR spectra (bottom) of compound **3** measured on a JEOL Eclipse+ 500 (^{1}H 500 MHz, ^{13}C 126 MHz). The peak at 1.56 ppm, marked by *, in the ^{1}H -NMR spectra stems from residues of water.

3. Fluorescence Lifetimes Measurements

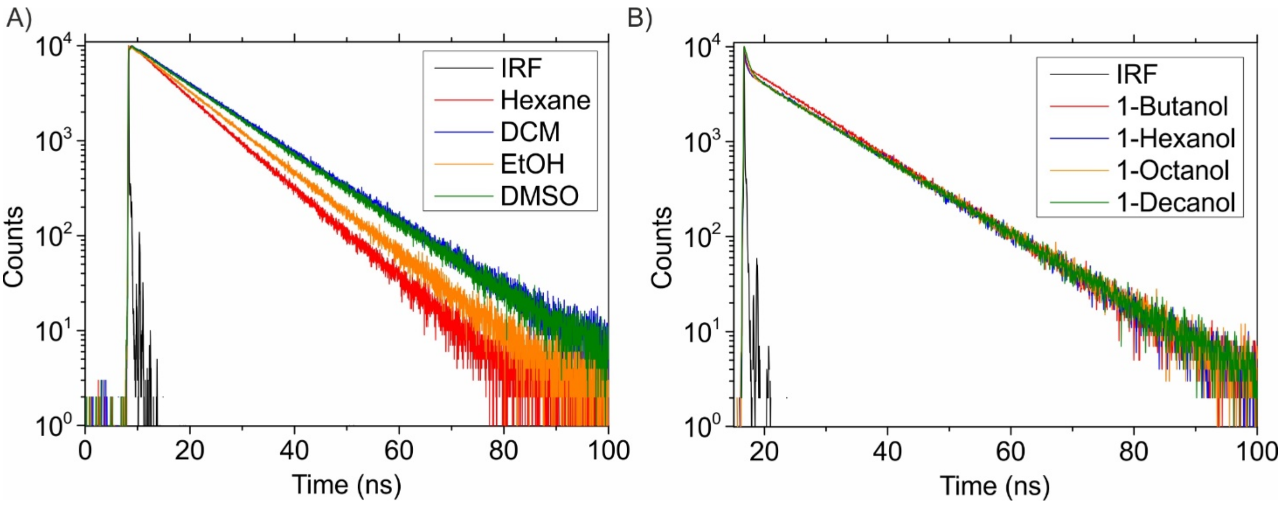

Figure S 3: Fluorescence lifetime decays of solutions of **1** obtained by time correlated single photon counting (TCSPC) with excitation at 375 nm. A) Hexane (red), DCM (blue), EtOH (orange) and DMSO (green). B) In primary alcohols of different chain length, 1-Butanol (red), 1-Hexanol (blue), 1-Octanol (orange) and 1-Decanol (green). The lifetime is irrespective of the chain length of the solvent alcohol relatively constant (see Table S2).

Table S 1: Mean fluorescence lifetimes of **1** in solvents of different polarity. Values for the normalized Dimroth-Reichardt Parameter E_T^N were taken from Ref.[16]

		**1**
E_T^N	Solvent	τ [ns]
0.006	n-hexane	8.92
0.309	DCM	11.97
0.444	DMSO	11.74
0.654	EtOH	10.06

4. Measurements in primary alcohols and mixtures of ethanol/polyethylene glycol

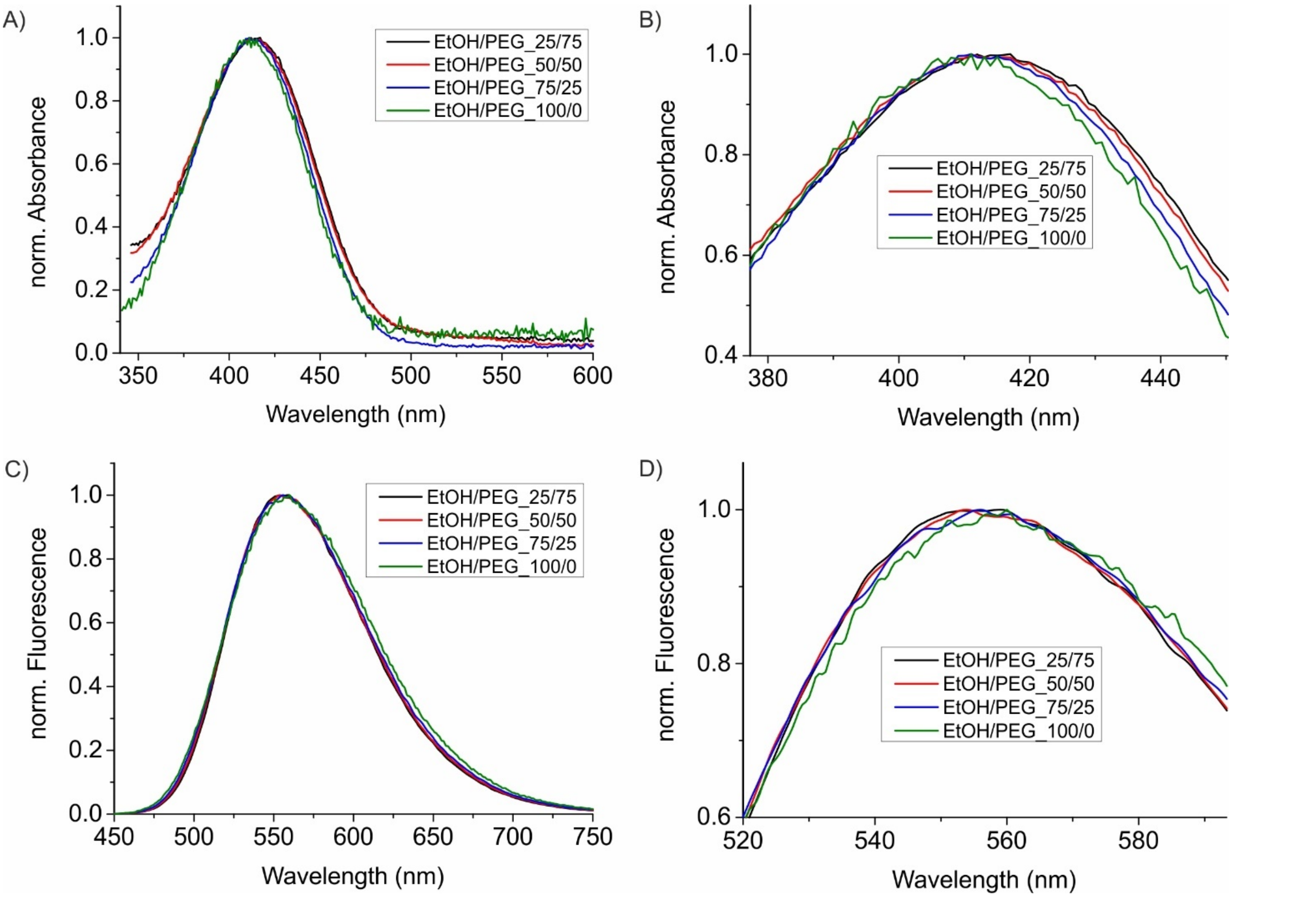

Figure S 4: Absorption spectra (A), zoomed Absorption spectra (B), Fluorescence spectra (C) and zoomed fluorescence spectra (D) of **1** in mixtures of ethanol/polyethylene glycol 25/75 (black), 50/50 (red), 75/25 (blue) and 100/0 (green).

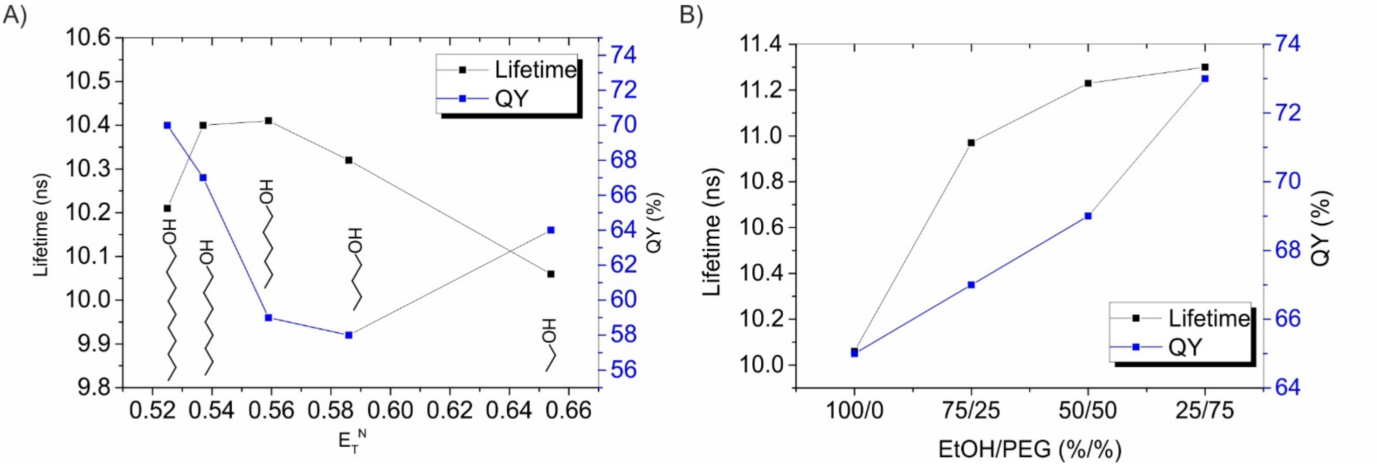

Figure S 5: A) Mean fluorescence lifetime values and fluorescence quantum yield of **1** vs. the normalized Dimroth-Reichardt Parameter E_T^N taken from Ref.[16] With increasing chain length of the alcohol, and thus decreasing polarity and increasing viscosity, the mean fluorescence lifetime and the fluorescence quantum yield slightly increase (values see **Table S3**). B) Mean fluorescence lifetime values and fluorescence quantum yield of **1** vs. the percentage of ethanol in mixtures of ethanol and polyethylengylcol (PEG). With increasing amount of PEG, thus increasing viscosity, the mean fluorescence lifetime, and the fluorescence quantum yield increase (values see **Table S4**).

Table S 2: Values of the viscosity, dielectric constant ε_r, the refractive index n and the resulting orientation polarizability of the five used primary alcohols taken from reference[17].

	Viscosity [mPa s]	ε_r	n	Δf
1-Decanol	11.9	8.1	1.437	0.205191892
1-Octanol	7.5	10.3	1.427	0.226258518
1-Hexanol	4.5	13.3	1.418	0.244370791
1-Butanol	3.0	17.85	1.399	0.264353401
Ethanol	1.1	24.3	1.35	0.292685944

Table S 3: Mean fluorescence lifetimes and fluorescence quantum yields of **1** in primary alcohols of different chain length. The % change was calculated with reference to ethanol. Values for the normalized Dimroth-Reichardt Parameter E_T^N were taken from Ref.[16]

		**1**			
E_T^N	Solvent	τ [ns]	% change	Φ [%]	% change
0.654	ethanol	10.06	0%	64	0%
0.586	1-butanol	10.32	2.6%	58	0%
0.559	1-hexanol	10.41	3.5%	59	0%
0.537	1-octanol	10.40	3.4	67	4.7%
0.525	1-decanol	10.21	1.5%	70	9.4%

Table S 4: Mean fluorescence lifetimes and fluorescence quantum yields of **1** in mixtures of EtOH/polyethylene glycole. The % change was calculated with reference to 100% ethanol. The E_T^N of PEG400 is 0.66. Thus, the polarity of the mixtures is barley changing. Values for the normalized Dimroth-Reichardt Parameter E_T^N were taken from Ref.[16, 18]

		**1**			
E_T^N	Solvent EtOH/PEG	τ [ns]	% change	Φ [%]	% change
0.654	100/0	10.06	0%	65	0%
	75/25	10.97	9.0%	67	3.0%
	50/50	11.23	11.6%	69	6.2%
	25/75	11.33	12.6%	73	12.3%

5. Lippert-Mataga Plot

A)

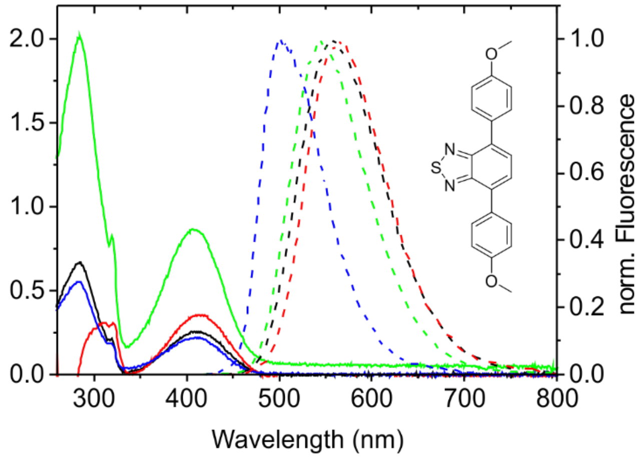

B)

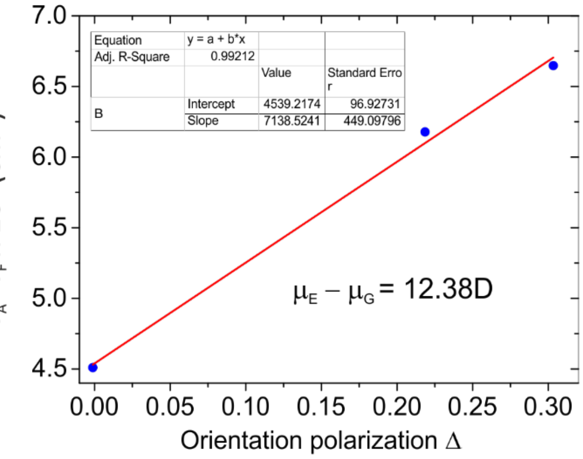

C)

1							
E_T^N	Solvent	Abs_{max}		Em_{max}		Stokes Shift	
		[nm]	[cm-1]	[nm]	[cm-1]	[nm]	[cm^{-1}]
0,009	hexane	411	2,43E+04	505	1,98E+04	94	4,53E+03
0,309	DCM	408	2,45E+04	546	1,83E+04	138	6,19E+03
0,444	DMSO	411	2,43E+04	566	1,77E+04	155	6,66E+03
0,654	ethanol	414	2,42E+04	560	1,79E+04	146	6,30E+03

Figure S 6: A) Absorption spectra (solid) and normalized fluorescence spectra (dashed) of **5** in solvents of different polarity. Hexane = blue, DCM = green, DMSO = red, black = EtOH with the molecular structure in the inset. B) Lippert-Mataga plot of **1** with ethanol left out due to its proticity. The fit data is given in the inset table. C) Absorption maxima, fluorescence maxima and stokes shift in wavelength and energy units for all four solvents ordered according to the normalized Dimroth-Reichardt Parameter E_T^N taken from Ref.[16]

6. Solid-state Fluorescence Measurements

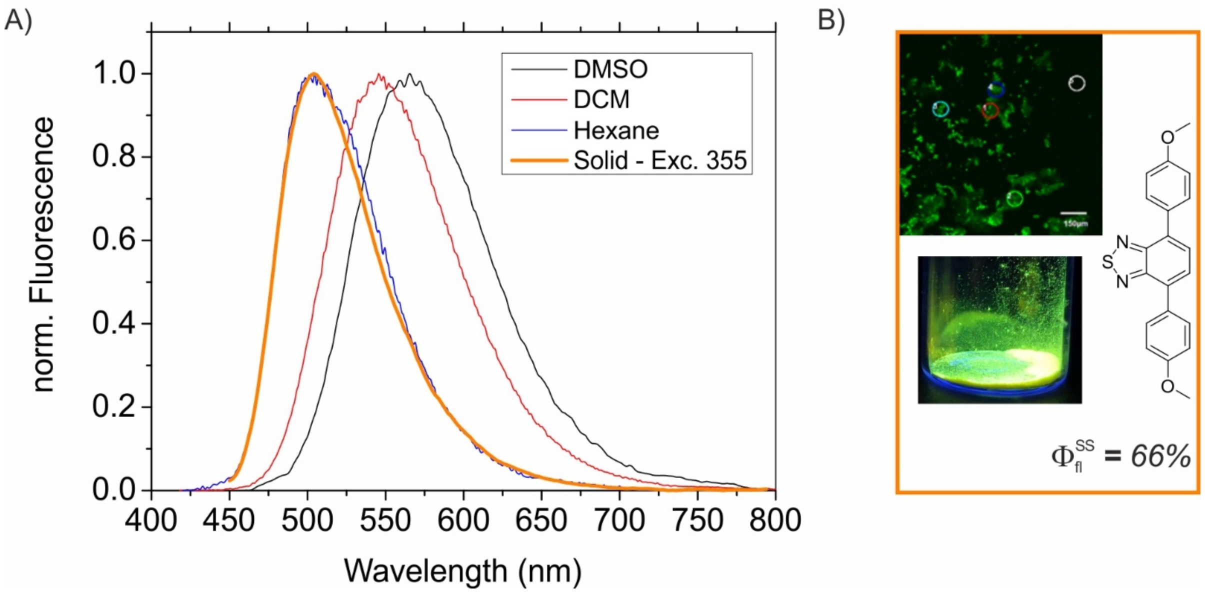

Figure S 7: A) Spatial resolved solid-state emission of **1** excited at 355 nm (orange, background corrected, smoothed, normalized) with the normalized fluorescence spectra in hexane (blue), DCM (red) and DMSO (black). B) Solid-state emission of ground crystals of **1** (Exc. 355 nm; Excitation DM BS20/80; UPLSAPO 10X NA:0.40) and photographs of the solid powder of **1** under radiation of the UV lamp at 366 nm. The solid-state fluorescence quantum yield is given in %.

7. Cyclic voltammetry measurements

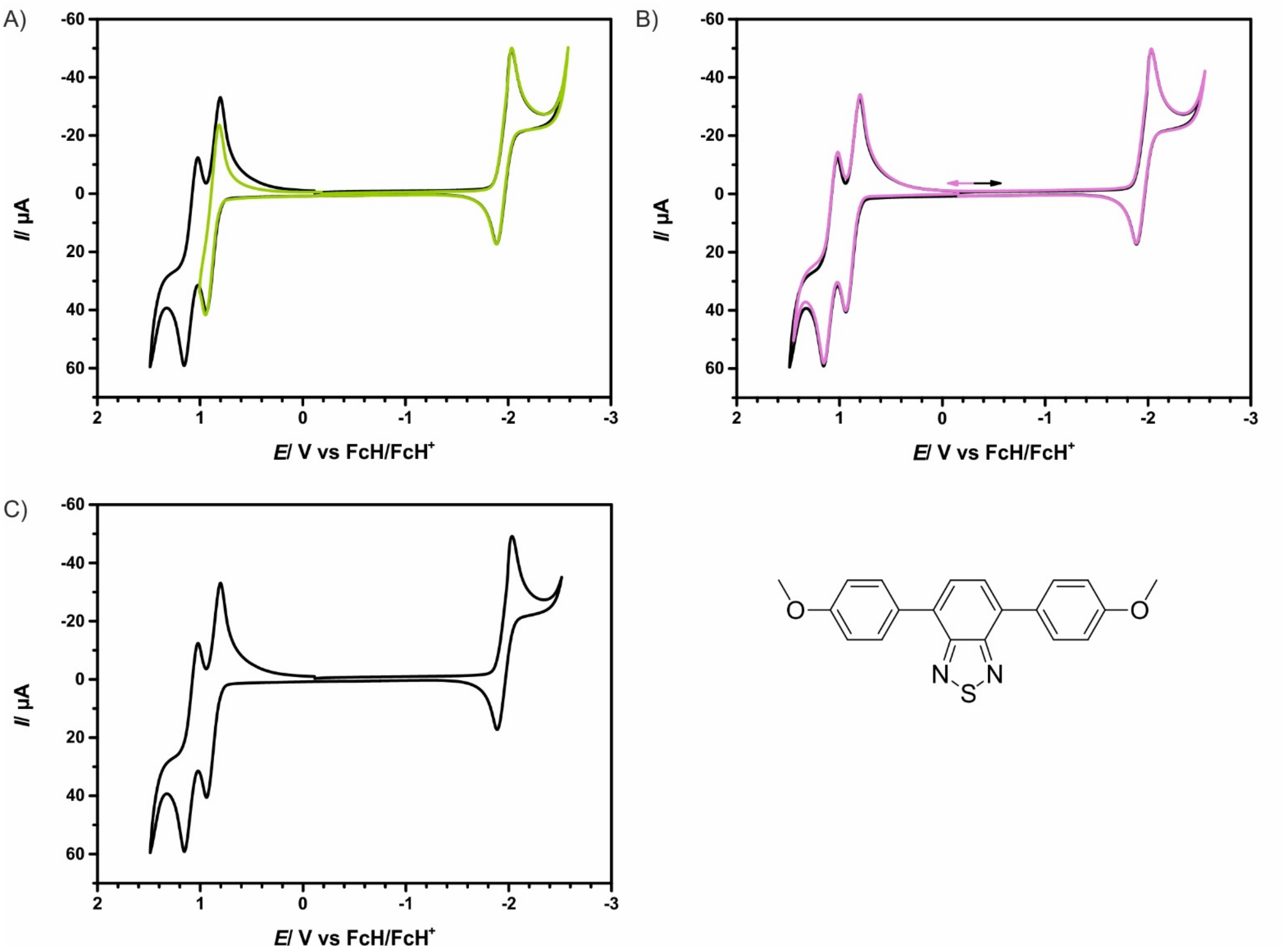

Figure S 8: Voltammograms of **1** measured at 100 mV/s measured in dichloromethane solutions with tetrabutylammonium hexafluorophosphate (0.1M). The arrows indicate the direction of measurement. A) Voltammogram measured from 1.3 V to -2.7 V (black) and from 1.0 V to -2.7 V (green) showing no remarkable difference in the visible processes. B) Comparison of measured directions, first oxidation (purple) and first reduction (black). There is no remarkable difference between the measurement directions. C) Voltammogram measured over the whole solvent window showing to reductions, an oxidation as well as a follow up process after the first cycle around 0 V.

Table S 5: Redox potentials E of **1** vs FcH/FcH+ measured in DCM / 0.1 NBu4PF6 at 100 mVs-1 at room temperature with a glassy carbon working electrode.

	E_1 (2nd Ox) / V	E_2 (1st Ox) / V	E_3 (1st Red) / V	E_4 (2nd Red) / V
1	+1.09	+0.87	-1.96	---

8. UV/Vis/NIR-spectroelectrochemistry

Table S 6: Absorption maxima in nm with the respective extinction coefficients of **1**. The absorption maxima and respective extinction coefficients for the reduced and oxidized species are given.

	λ / nm (e /10^3 M^{-1} cm^{-1})
**1**	285 (20161), 408 (8611)
**1^+**	244 (8473), 262 (8732), 309 (7946), 318 (7971), 350 (8521), 395 (6453), 531 (12037), 666 (11331), 832 (11943), 980 (8536) sh, 1124 (18147)
**1^{2+}**	228 (8732) 303 (9836), 388 (4391) sh, 403 (5349), 654 (50248)
**1^-**	237 (16281), 283 (14701), 320 (9461) sh, 408 (3368), 767 (1036) br, 1246 (846) br

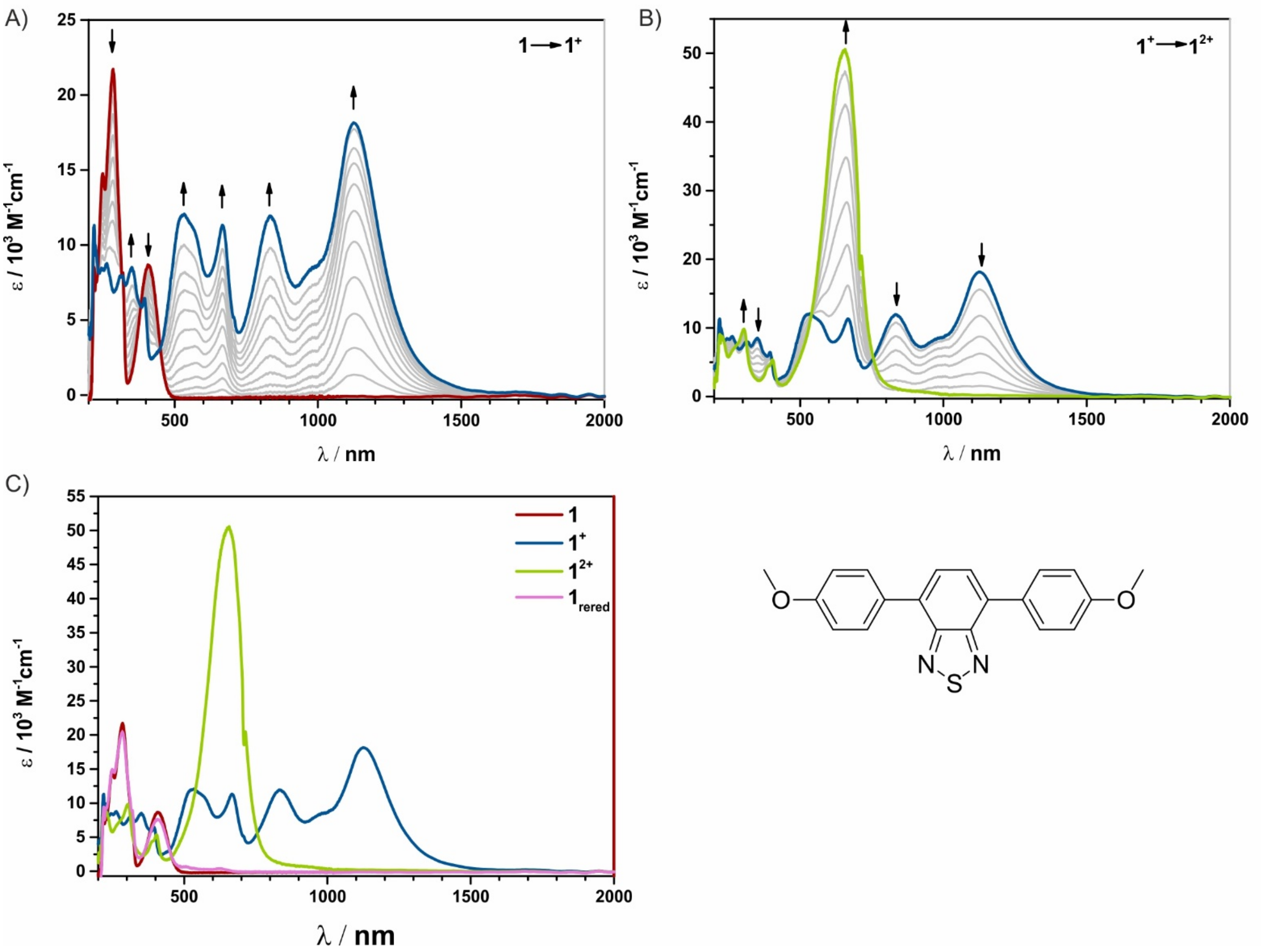

Figure S 9: Absorption spectra of **1** and resulting species upon oxidation. A) Red is the absorption spectra of **1** in its neutral form, blue the absorption spectra upon first oxidation. B) Absorption of **1^+** (blue) and after second oxidation to **1^{2+}** (green). C) Absorption of neutral **1** (red), **1^+** (blue), **1^{2+}** (green) and re-reduced **1**. The resemblance of the red and the pink curves show the reversibility of the redox process.

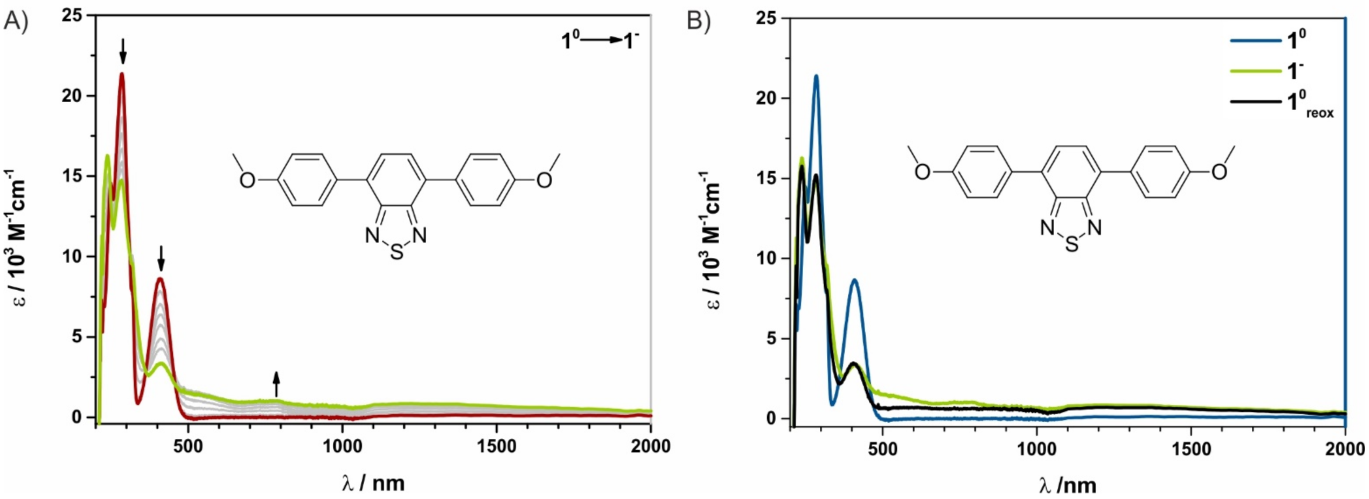

Figure S 10: Absorption spectra of **1** and resulting species upon reduction. A) Red is the absorption spectra of **1** in its neutral form, green the absorption spectra upon first reduction. B) Absorption of **1** (blue) and after first reduction to **1**$^-$ (green) and after re-oxidation (black). The re-oxidation (blue to black) is not complete.

9. EPR spectroelectrochemistry of **1**

Table S 7: Overview of nuclei used for the EPR simulation of **1** along with hyperfine coupling constants in MHz and mT.

No. of nuclei and nuclei	A / MHz	A / mT
2x N	1.85	0.07
2x H	3.7	0.13
2x H	5.55	0.19
2x H	1.85	0.06
4x H	2.78	0.09

g_{sim} = 2.0028

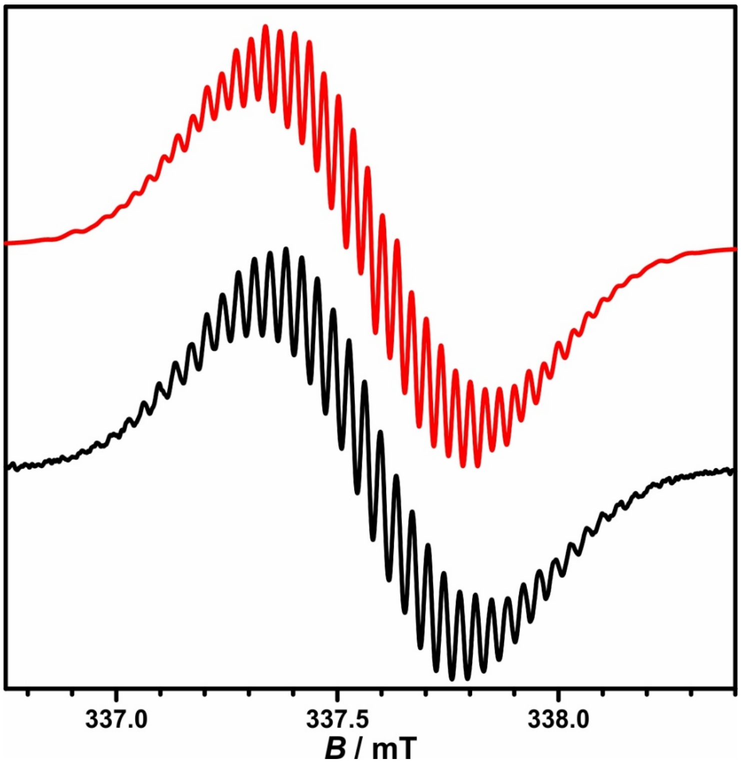

Figure S 11: Measured (black) and simulated (red) EPR spectra of **1**.

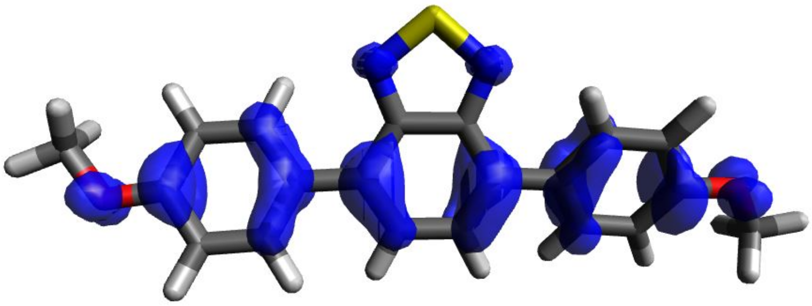

Figure S 12: Spin density of **1** calculated at the PBE0/IGLO-III level.

10. TD-DFT Calculations for 1 (singlet)

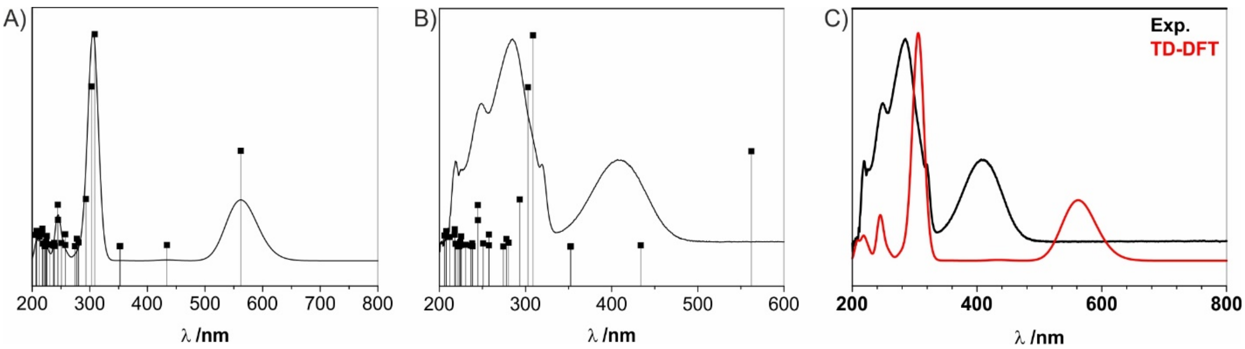

Figure S 13: Calculated TD-DFT spectrum with discrete transitions (A), experimental spectrum with discrete calculated transitions (B) and experimental (black) and calculated TD-DFT spectrum (red) (C).

Table S 8: TD-DFT transitions for **1**.

State	Difference density (iso value 0.002)	Transition homo lumo	Calculated Transition energy	Oscillator strength	Exp. transition energy	Molar absorption coefficient 10^3
1	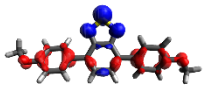	HOMO -> LUMO (0.98)	562.2	0.2213	408	8.6
2	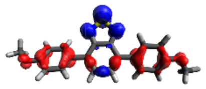	HOMO-1 -> LUMO (0.98)	433.9	0.0028		
4	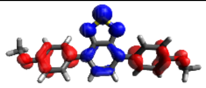	HOMO-3 -> LUMO (0.95)	352.6	0.0008		
5	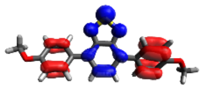	HOMO-4 -> LUMO (0.34)<br>HOMO -> LUMO+1 (0.58)	308.8	0.4912	285	20.1

Table S 9: Selected molecular orbitals for **1**.

HOMO-4	HOMO-3	HOMO-1	HOMO	LUMO	LUMO+1
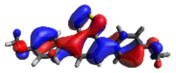	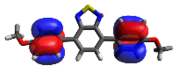	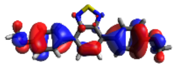	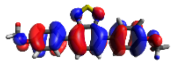	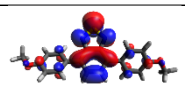	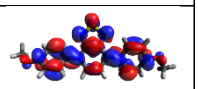

11. TD-DFT for 1^+ (doublet)

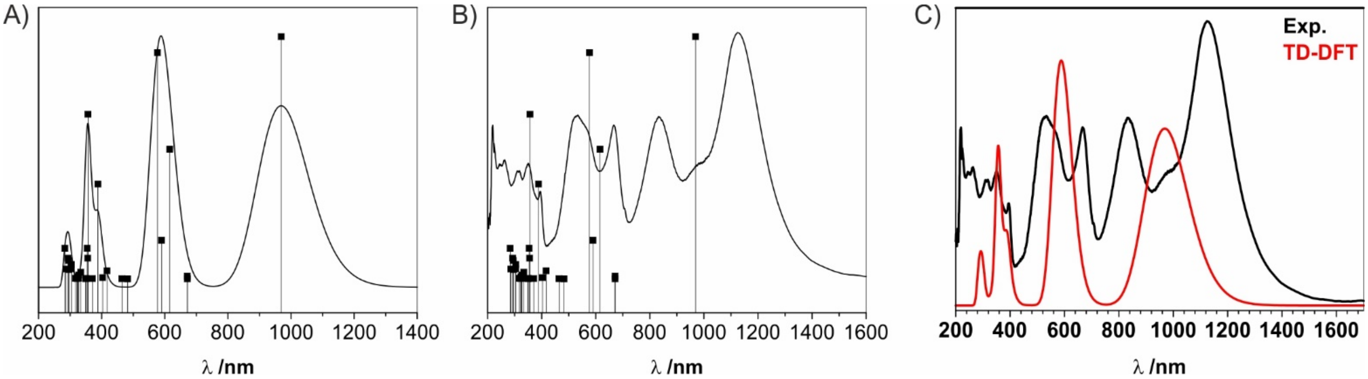

Figure S 14: Calculated TD-DFT spectrum of **1**⁺ with discrete transitions (left), experimental spectrum with discrete calculated transitions (middle) and experimental (black) and calculated TD-DFT spectrum (red) (left).

Table S 10: TD-DFT transitions for 1⁺.

State	Difference density (iso value 0.002)	Transition homo lumo	Calculated Transition energy	Oscillator strength	Exp. transition energy	Molar absorption coefficient 10^3
1	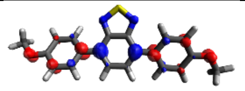	HOMOβ -> LUMOβ (0.87)	969.0	0.3289	1124	18.1
4	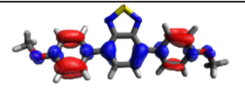	HOMOα -> LUMOα (0.28)<br>HOMOβ-4 -> LUMOβ (0.57)	615.9	0.1760	666	11.3
6	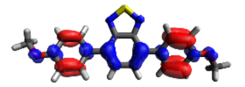	HOMOα -> LUMOα (0.43)<br>HOMOβ-4 -> LUMOβ (0.40)	577.0	0.3074	531	12.0
11	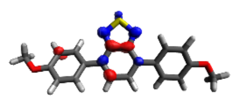	HOMOα-5 -> LUMOα+1 (0.25)<br>HOMOβ-1 -> LUMOβ+1 (0.26)	388.3	0.1287	395	6.4
13	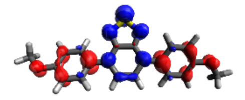	HOMOα -> LUMOα+1 (0.51)<br>HOMOβ-7 -> LUMOβ (0.27)	356.7	0.2236	350	8.5

Table S 11: Selected molecular orbitals for 1⁺.

HOMOβ-7	HOMOβ-4	HOMOβ-3	HOMOβ-2	HOMOβ-1	HOMOβ
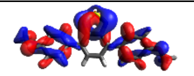	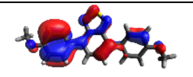	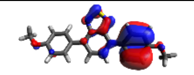	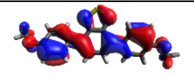	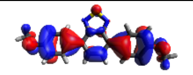	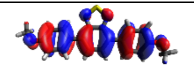
LUMOβ	LUMOβ+1	HOMOα-5	HOMOα	LUMOα	LUMOα+1
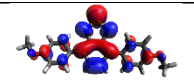	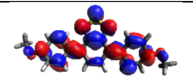	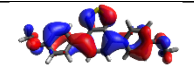	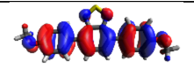	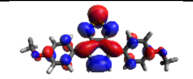	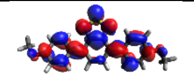

Table S1: Orbital energies for 1^{+} (doublet state).

12. TD-DFT for 1^{2+} (singlet)

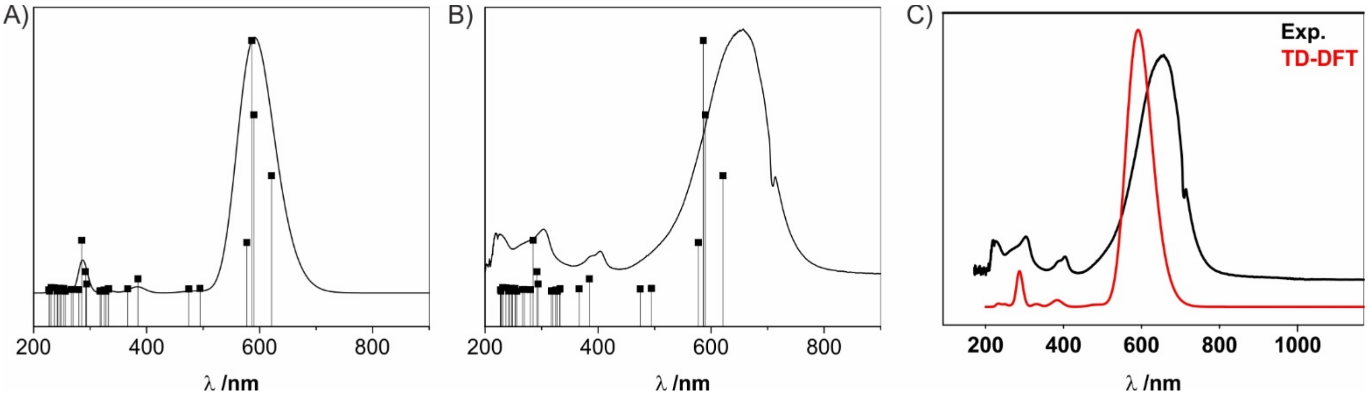

Figure S 15: Calculated TD-DFT spectrum of **1**$^{2+}$ with discrete transitions (left), experimental spectrum with discrete calculated transitions (middle) and experimental (black) and calculated TD-DFT spectrum (red) (left).

Table S 12: TD-DFT transitions for **1**$^{2+}$.

State	Difference density (iso value 0.002)	Transition homo lumo	Calculated Transition energy	Oscillator strength	Experimental transition energy	Molar absorption coefficient 10^3
1	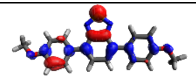	HOMO -3-> LUMO (0.12)<br>HOMO -1-> LUMO (0.58)<br>HOMO -> LUMO (0.23)	621.0	0.2998	654	50.2
2	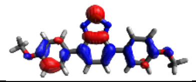	HOMO -2-> LUMO (0.45)<br>HOMO -> LUMO (0.42)	586.2	0.6512		
3	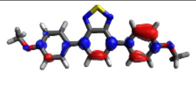	HOMO -4-> LUMO (0.20)<br>HOMO -2-> LUMO (0.32)<br>HOMO -1-> LUMO (0.19)<br>HOMO -> LUMO (0.25)	589.7	0.4584		
4	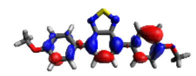	HOMO -4-> LUMO (0.31)<br>HOMO -3-> LUMO (0.55)	577.3	0.1263		
7	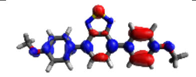	HOMO-6 -> LUMO (0.13)<br>HOMO -> LUMO+1 (0.65)	384.7	0.0319	403	5.3
8	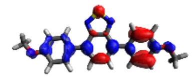	HOMO-7 -> LUMO (0.12)<br>HOMO-6 -> LUMO (0.74)	366.5	0.0060	388	4.3
17	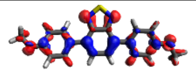	HOMO -5-> LUMO+1 (0.18)<br>HOMO -4-> LUMO+1 (0.39)<br>HOMO -3-> LUMO+1 (0.18)	285.0	0.1321	303	9.8

Table S 13: Selected molecular orbitals for **1**$^{2+}$.

HOMO-7	HOMO-6	HOMO-5	HOMO-4	HOMO-3
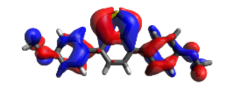	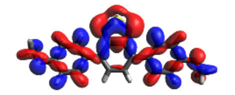	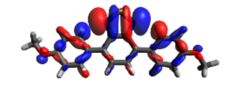	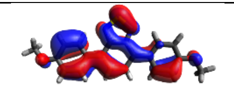	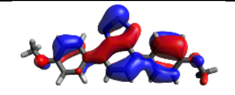
HOMO-2	HOMO-1	HOMO	LUMO	LUMO+1
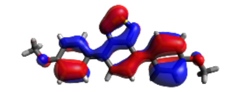	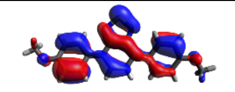	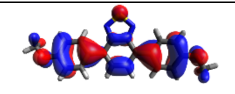	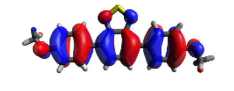	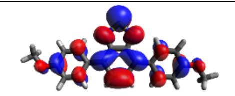

13. TD-DFT for 1^{+} with explicit solvent molecules

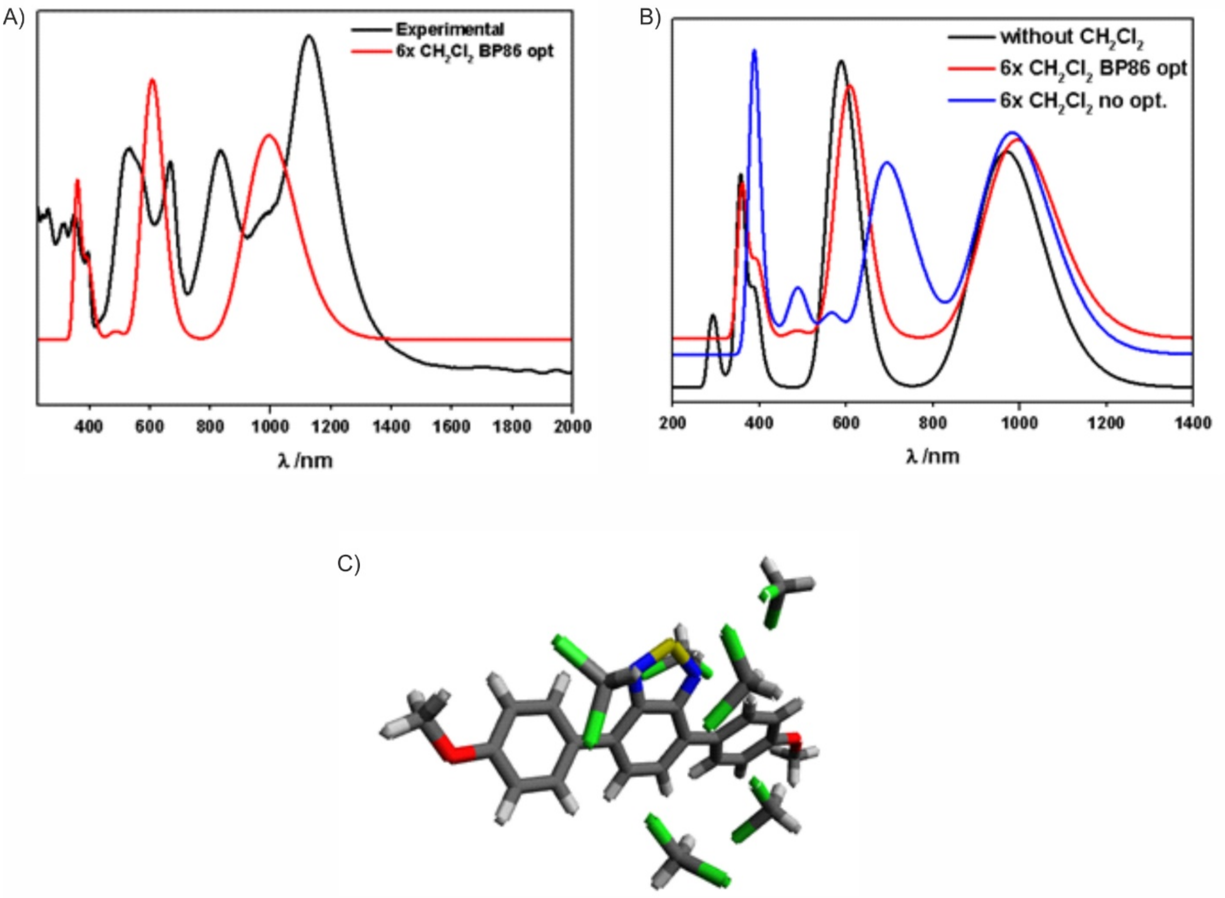

Figure S 16: A) Experimental (black) and calculated (red) TD-DFT spectrum of **1⁺** with six dichloromethane molecules B) Comparison of the three calculated TD-DFT spectra without solvent (black), with solvent molecules and optimized with BP86 (red) and without optimization (blue). C) Optimized structure of **1⁺** with six dichloromethane molecules as solvent.

14. TD-DFT for 1⁻ (doublet)

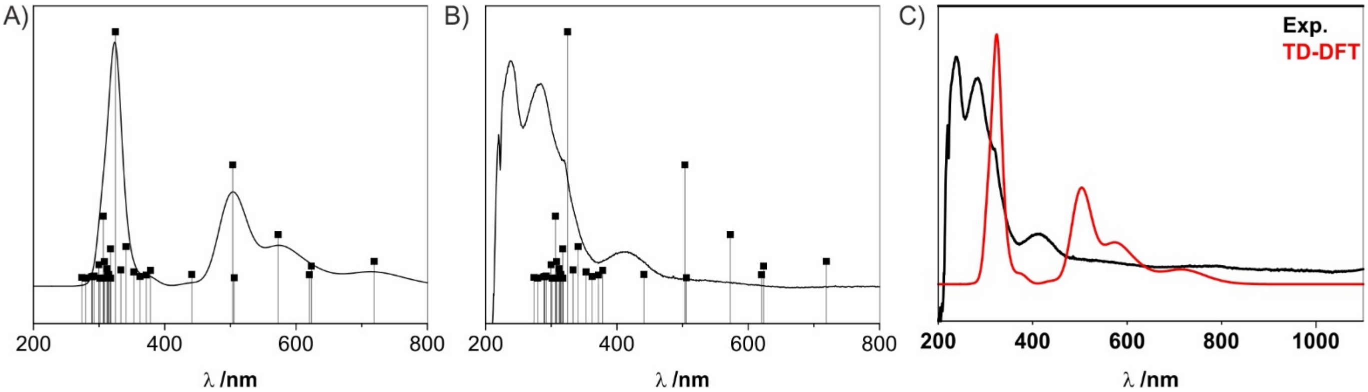

Figure S 17: Calculated TD-DFT spectrum with discrete transitions (left), experimental spectrum with discrete calculated transitions (middle) and experimental (black) and calculated TD-DFT spectrum (red) (left).

Table S 14. TD-DFT transitions for **1**⁻.

State	Difference density (iso value 0.002)	Transition homo lumo	Calculated Transition energy	Oscillator strength	Exp. transition energy	Molar absorption coefficient 10^3
3	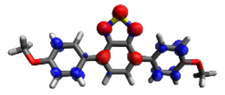	HOMOα -> LUMOα +1(0.26)<br>HOMOβ -> LUMOβ (0.52)	572.7	0.0885	767	1.0
4	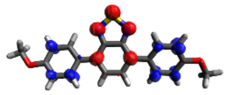	HOMOα -> LUMOα +2(0.86)	623.3	0.0245		
5	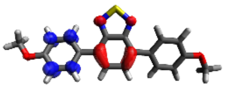	HOMOα -> LUMOα +3(0.90)	503.5	0.2302	408	3.3
13	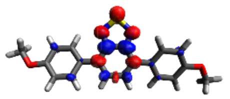	HOMOα-1 -> LUMOα (0.34)<br>HOMOβ -> LUMOβ+1(0.33)	324.9	0.5005	283	14.7

Table S 15: Selected molecular orbitals for **1**⁻.

HOMOα-1	HOMOα	LUMOα	LUMOα+1	LUMOα+2
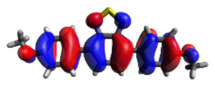	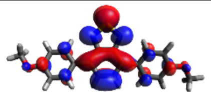	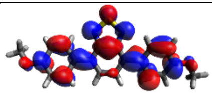	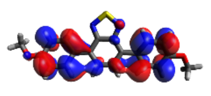	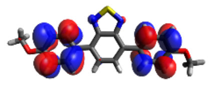
LUMOα+3	HOMOβ-1	HOMOβ	LUMOβ	LUMOβ+1
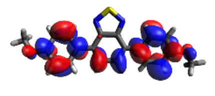	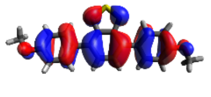	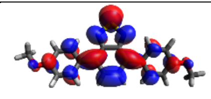	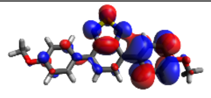	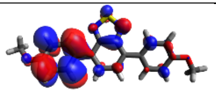

15. In situ fluorescence measurements during oxidation and re-reduction

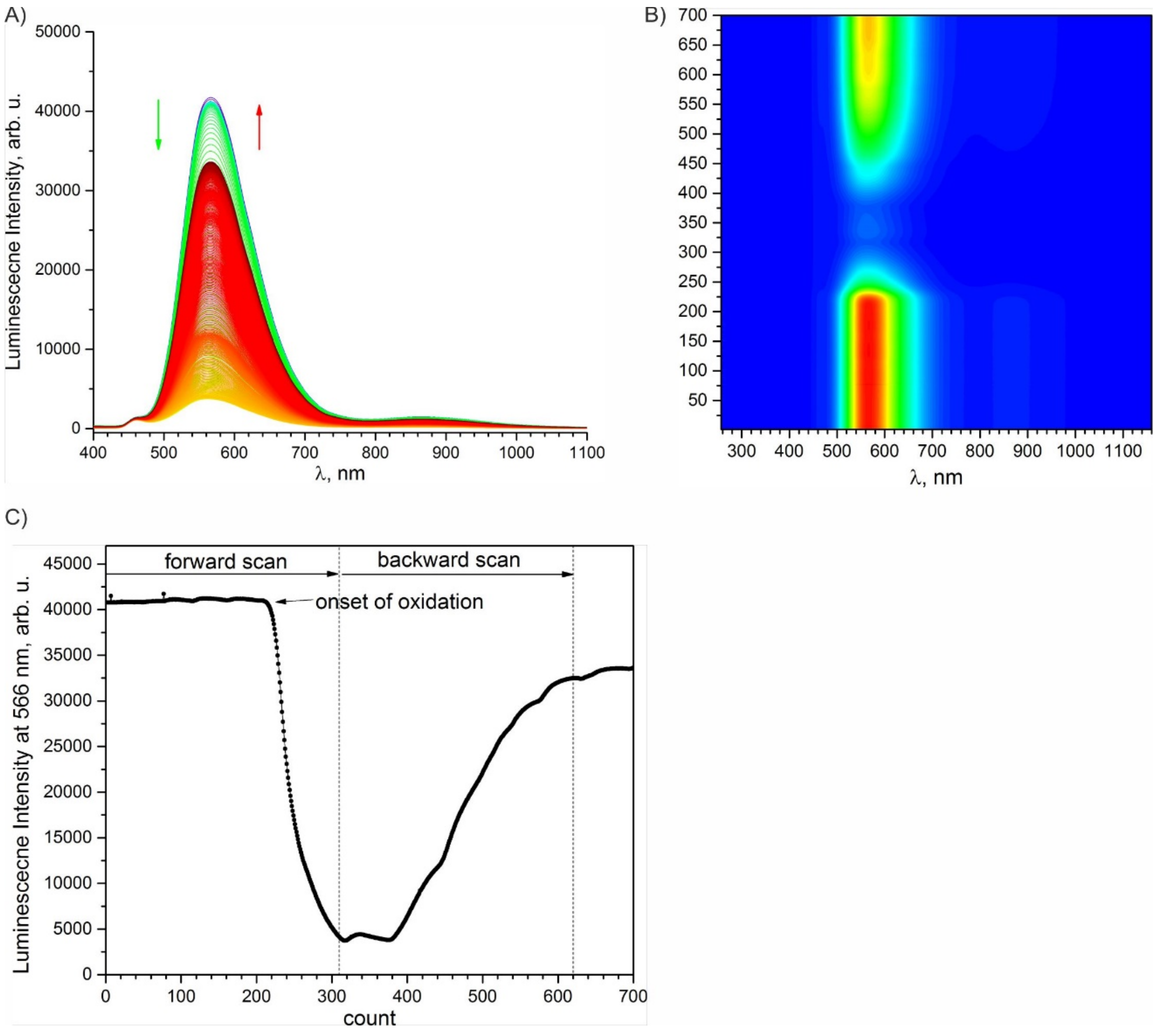

Figure S 18: (A) Fluorescence spectra measured in situ during CV at two oxidation steps, CV was swept with 5 mV/sec, accumulation of one fluorescence spectrum took 1 second, totally 700 spectra were measured. No changes in the spectral shape were observed, only the variation of intensity scale. (B) 2D contour plot – a strong decay of fluorescence can be seen when the oxidation is started, followed by a slow restoration of the intensity upon re-reduction during a backward scan. (C) Profile of the intensity at 566 nm (maximum of the fluorescence). Fluorescence was excited with 405 nm laser, the spectra were measured with a long-pass filter at 450 nm on the detector side (i.e. light at shorter wavelength could not go through).

16. Dilution series of compound 1 in EtOH and DCM

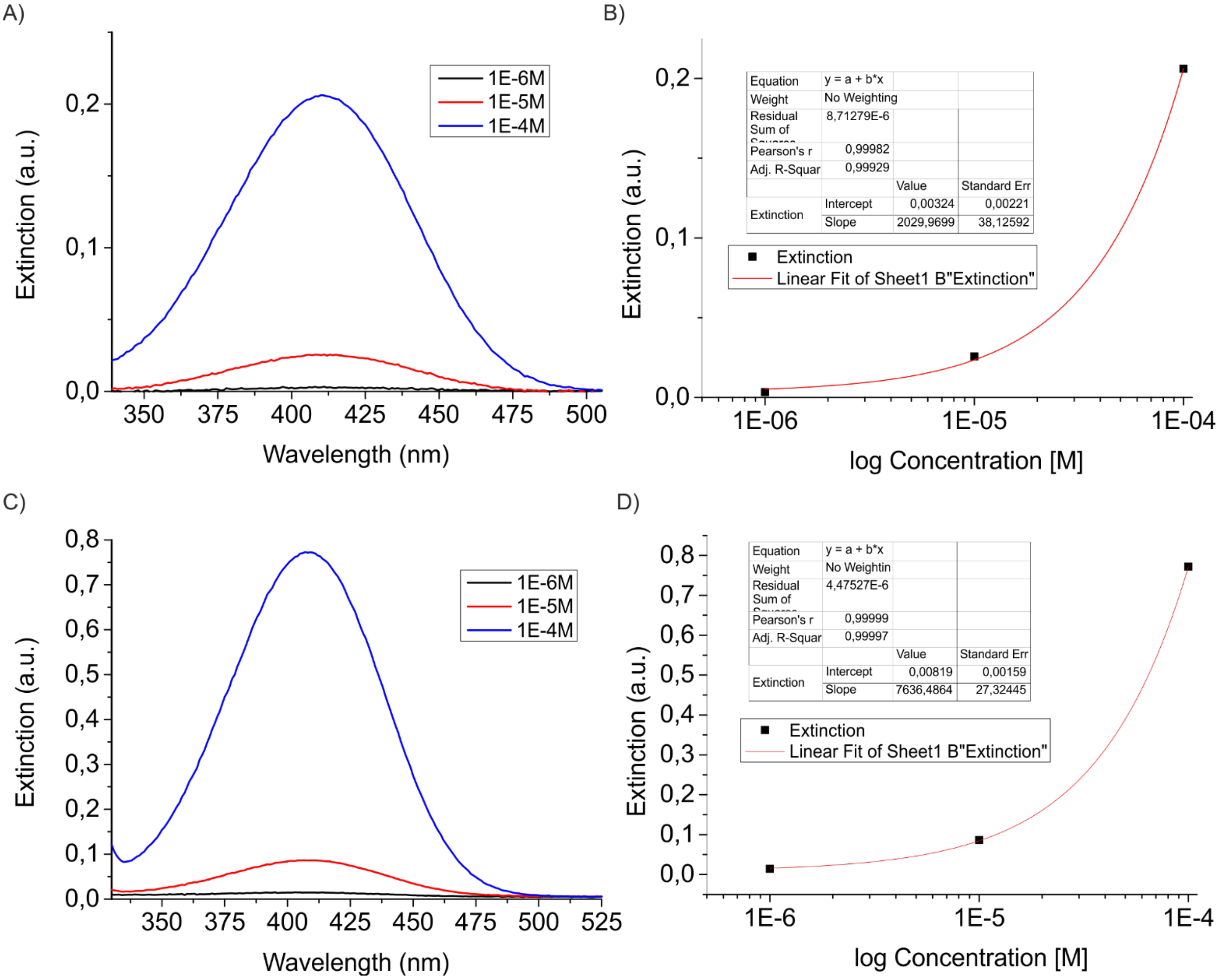

Figure S 19: A) Extinction of compound **1** in EtOH at different concentrations given in the inset. B) Plot of extinction versus the concentration of the respective curve given in plot A. The concentration is given on a logarithmic scale, the linear fit shows that the compound fulfils the lambert-beer law. C) Extinction of compound **1** in DCM at different concentrations given in the inset. D) Plot of extinction versus the concentration of the respective curve given in plot C. The concentration is given on a logarithmic scale, the linear fit shows that the compound fulfils the Lambert-Beer law.

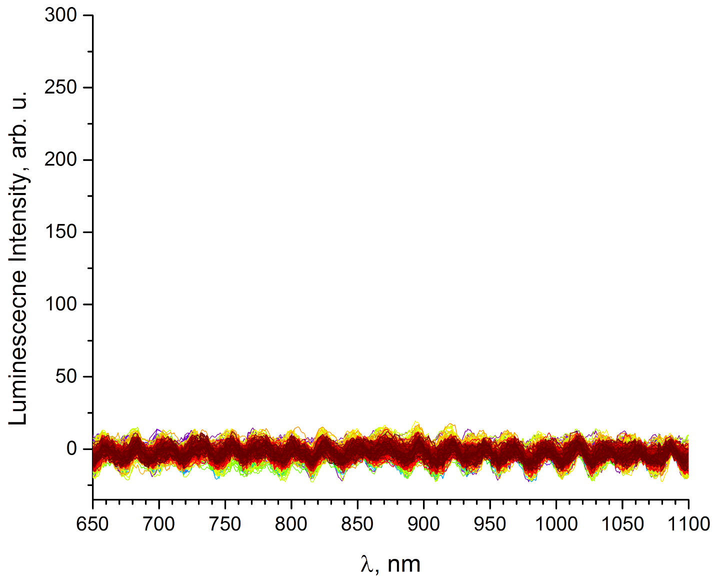

Figure S 20: Fluorescence spectra measured in situ during CV at two oxidation steps, all conditions are the same as in the previous page, but with the excitation at 638 nm and with a long-pass filter at 700 nm. No signal appears in the NIR range. Note also that intensity scale here is much smaller than on the previous page, and that the laser power was 0.5 mW on the previous page, and was up to 100 mW for the measurements with 638 nm laser.

[1] a) M. Krejčik, M. Daněk, F. Hartl, *Journal of Electroanalytical Chemistry and Interfacial Electrochemistry* **1991**, *317*, 179; b) J. Klein, A. Stuckmann, S. Sobottka, L. Suntrup, M. van der Meer, P. Hommes, H. U. Reissig, B. Sarkar, *Chem. Eur. J.* **2017**, *23*, 12314.
[2] F. Neese, *Wiley Interdisciplinary Reviews: Computational Molecular Science* **2018**, *8*.
[3] S. Grimme, J. G. Brandenburg, C. Bannwarth, A. Hansen, *J. Phys. Chem.* **2015**, *143*.
[4] a) A. D. Becke, *J. Phys. Chem.* **1993**, *98*, 5648; b) C. Lee, W. Yang, R. G. Parr, *Phys. Rev. B Condens. Matter.* **1988**, *37*, 785.
[5] a) S. Grimme, S. Ehrlich, L. Goerigk, *J. Comput. Chem.* **2011**, *32*, 1456; b) S. Grimme, J. Antony, S. Ehrlich, H. Krieg, *The Journal of chemical physics* **2010**, *132*, 154104; c) S. Grimme, *J. Comput. Chem.* **2004**, *25*, 1463; d) S. Grimme, *J. Comput. Chem.* **2006**, *27*, 1787; e) A. D. Becke, *Phys. Rev. A* **1988**, *38*, 3098; f) J. P. Perdew, *Phys. Rev. B* **1986**, *33*, 8822; g) J. P. Perdew, *Phys. Rev. B* **1986**, *34*, 7406.
[6] C. van Wüllen, *J. Phys. Chem.* **1998**, *109*, 392.
[7] D. A. Pantazis, X.-Y. Chen, C. R. Landis, F. Neese, *J. Chem. Theory Comput.* **2008**, *4*, 908.
[8] a) F. Neese, *J. Comput. Chem.* **2003**, *24*, 1740; b) F. Neese, F. Wennmohs, A. Hansen, U. Becker, *Chem. Phys.* **2009**, *356*, 98; c) O. Vahtras, J. Almlöf, M. W. Feyereisen, *Chem. Phys. Lett.* **1993**, *213*, 514; d) J. L. Whitten, *J. Phys. Chem.* **1973**, *58*, 4496; e) R. Izsák, F. Neese, *J. Phys. Chem.* **2011**, *135*; f) F. Neese, G. Olbrich, *Chem. Phys. Lett.* **2002**, *362*, 170; g) T. Petrenko, S. Kossmann, F. Neese, *J. Phys. Chem.* **2011**, *134*.
[9] a) K. Eichkorn, F. Weigend, O. Treutler, R. Ahlrichs, *Theoretical Chemistry Accounts: Theory, Computation, and Modeling (Theoretica Chimica Acta)* **1997**, *97*, 119; b) K. Eichkorn, O. Treutler, H. Öhm, M. Häser, R. Ahlrichs, *Chem. Phys. Lett.* **1995**, *242*, 652.
[10] V. Barone, M. Cossi, *J. Phys. Chem. A* **1998**, *102*, 1995.
[11] P. O. Löwdin, *J. Phys. Chem.* **1950**, *18*, 365.
[12] a) *Avogadro: an open-source molecular builder and visualization tool. Version 1.2.0. modified version with extended ORCA support* *http://avogadro.openmolecules.net/ and https://orcaforum.cec.mpg.de/*; b) M. D. Hanwell, D. E. Curtis, D. C. Lonie, T. Vandermeersch, E. Zurek, G. R. Hutchison, *J. Cheminform* **2012**, *4*, 17.
[13] W. Kutzelnigg, U. Fleischer, M. Schindler, *NMR: Basic Principles and Progress*, Springer-Verlag, Berlin, **1990**.
[14] B. A. DaSilveira Neto, A. S. A. Lopes, G. Ebeling, R. S. Gonçalves, V. E. U. Costa, F. H. Quina, J. Dupont, *Tetrahedron* **2005**, *61*, 10975.
[15] K.-W. Park, L. A. Serrano, S. Ahn, M. H. Baek, A. A. Wiles, G. Cooke, J. Hong, *Tetrahedron* **2017**, *73*, 1098.
[16] C. Reichardt, *Chem. Rev.* **1994**, *94*, 2319.
[17] a) J. R. Lakowicz, *Principles of Fluorescence Spectroscopy*, **2006**; b) J. Ortega, *J. Chem. Eng. Data* **1982**, *27*, 312; c) B. García, R. Alcalde, S. Aparicio, J. M. Leal, *Phys. Chem. Chem. Phys.* **2002**, *4*, 1170.
[18] A. R. Harifi-Mood, M. Abbasi, *J. Solution Chem.* **2018**, *47*, 1503.
